# Polarity and chirality control of an active fluid by passive nematic defects

**DOI:** 10.1038/s41563-022-01432-w

**Published:** 2022-12-30

**Authors:** Alfredo Sciortino, Lukas J. Neumann, Timo Krüger, Ivan Maryshev, Tetsuhiko F. Teshima, Bernhard Wolfrum, Erwin Frey, Andreas R. Bausch

**Affiliations:** 1 Lehrstuhl für Zellbiophysik E27, Technische Universität München, Garching, Germany; 2 Center for Functional Protein Assemblies, Garching bei München, Germany; 3 Arnold Sommerfeld Center for Theoretical Physics (ASC) and Center for NanoScience (CeNS), Department of Physics, Ludwig-Maximilians-Universität, München, Germany; 4 Neuroelectronics, Department of Electrical Engineering, Technische Universität München, Garching, Germany; 5 Medical & Health Informatics Laboratories, NTT Research Incorporated, Sunnyvale, CA USA; 6 Matter to Life Program, Max Planck School, München, Germany; 7 Center for Organoid Systems and Tissue Engineering (COS), Technische Universität München, Garching, Germany

**Keywords:** Biological physics, Motility, Liquid crystals, Bioinspired materials

## Abstract

Much like passive materials, active systems can be affected by the presence of imperfections in their microscopic order, called defects, that influence macroscopic properties. This suggests the possibility to steer collective patterns by introducing and controlling defects in an active system. Here we show that a self-assembled, passive nematic is ideally suited to control the pattern formation process of an active fluid. To this end, we force microtubules to glide inside a passive nematic material made from actin filaments. The actin nematic features self-assembled half-integer defects that steer the active microtubules and lead to the formation of macroscopic polar patterns. Moreover, by confining the nematic in circular geometries, chiral loops form. We find that the exact positioning of nematic defects in the passive material deterministically controls the formation and the polarity of the active flow, opening the possibility of efficiently shaping an active material using passive defects.

## Abstract (web summary)

Defects of a passive nematic liquid crystal made from actin filaments pattern the collective behaviour of active microtubules, creating macroscopic polar patterns and chiral loops.

## Main

The macroscopic characteristics of materials can depend on microscopic impurities they contain. For instance, defects in the crystalline order of materials strongly affect their mechanical or transport properties^1^. Controlling defects, a fundamental way to manipulate materials, is also starting to find applications in the field of soft matter^2^. Active materials, composed of microscopic components able to turn energy into motion, are similarly often characterized in terms of the presence of defects and their dynamics^3,4^. Defects in the alignment of active elongated particles have been identified in a plethora of different contexts^5^ and for instance play a role in cytoskeletal self-organization^6–9^, cell motion^10–12^ and biological development^13,14^. Hence, one promising way to control active matter is controlling the system’s boundary condition or topology to control defects^7,15–19^. In these cases, however, defects are themselves part of the active system and thereby move or spontaneously form and annihilate, so that only limited control of their position, number and dynamics is possible.

A different strategy is to embed active systems into a passive medium, taking advantage of our ability to control traditional materials. Passive material properties and their defects have indeed been shown to influence the emergence of collective structures^8,20–24^. For instance, nematic defects can induce distortions in a passive material that will shape the behaviour of the active system they contain in a non-trivial way^25,26^. Confining swimming bacteria in a liquid crystal has indeed shed light on the interplay between active matter and passive defects, showing these latter can be used to shape pattern formation^27–30^. However, most of these results lack microscopic resolution and are affected by long-range hydrodynamic interactions that might overshadow local microscopic behaviours. To understand the potential of passive nematic materials to control active systems, microscopic resolution of the interactions between the two is needed.

Here we steer the pattern formation process of an active system by the presence of a passive liquid crystal. This is achieved by coupling a two-dimensional microtubule (MT) gliding assay to a self-assembled passive actin nematic featuring half-integer nematic defects. We observe that under these conditions, gliding filaments form ordered, polar structures. We image with high resolution their emergence and pinpoint nematic distortions originating from defects as their source. Specifically, −1/2 defects induce long-range distortions in the material that affect the active flow. More strikingly, the conformation of +1/2 defects is instead found to be a funnelling and polarity-sorting element^9^. Overall, this leads to the emergence of polar active flow, despite the nematic symmetry of the passive material. In addition, controlling the total nematic charge by confining the system turns polar streams into chiral loops. The formation of patterns and their shape are solely consequences of the shape of the passive nematic. It is indeed the precise positioning in space of point defects that steers the active fluid flow on a larger scale, an observation we can rationalize by simulations, fully recapturing experimental results.

## Microtubules inside a nematic assemble into polar streams

**Fig. 1:**
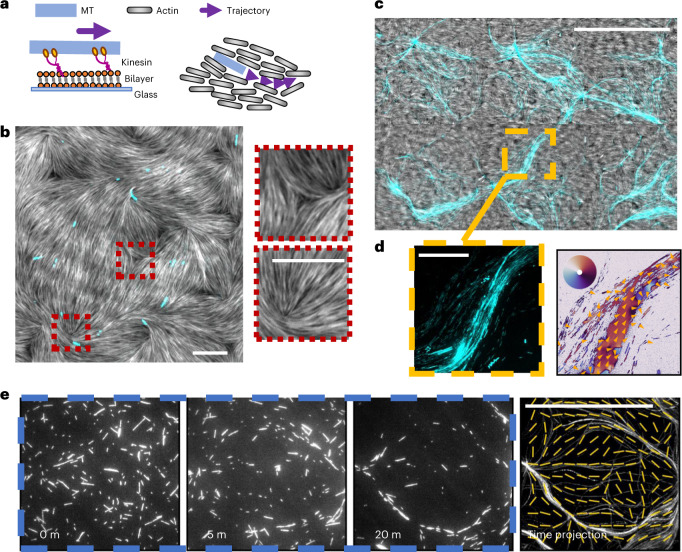
Description of the system and formation of polar streams. **a**, Schematic of the system. Kinesin motors are bound to a SLB and propel short, stabilized MTs. A passive actin nematic is sedimented on top, and MTs glide inside it. **b**, Microscopy image of the assembled nematic (coloured in grey) containing MTs (cyan). The nematic quickly assembles and features topological defects of half-integer charge, marked in the image in red and enlarged in the insets. MTs align and glide within the nematic. MT density is *σ* = 0.003 MTs µm^–2^. Scale bars, 10 µm. **c**, Over time, the MTs assemble into dense polar streams (cyan) with a size much bigger than that of individual filaments. MT density is *σ* = 0.08 MTs µm^–2^. Scale bar, 100 µm. **d**, Detail of a stream (left) and mean flow of the MTs (right). MT streams are found to be locally polar, with MTs mostly gliding in the same direction as shown by the mean optical flow colour-coded by the local orientation (Methods). Orange arrows display the flow direction. The orientation colour scale is shown in the inset coloured wheel. Scale bar, 50 µm. **e**, An initial isotropic distribution of MTs (white) evolves into streams as the nematic sediments. The last picture on the right shows a maximum intensity time projection (between 20 and 30 minutes) with an overlay of the final actin nematic director field (yellow), showing how formed streams move along the nematic director field. Scale bar, 100 µm.

Our experimental set-up consists of short (~2 µm) fluorescent MTs, processively propelled by streptavidin-tagged kinesin motors bound to a fluid, supported lipid bilayer (SLB) containing biotin. Moreover, 1 µM of short (~0.8 µm), fluorescent actin filaments together with a depletant are also present (Fig. 1a, Methods and Supplementary Information). Under these conditions, the actin filaments quickly assemble into a two-dimensional nematic^31,32^, featuring both +1/2 and −1/2 nematic defects (Fig. 1b, Supplementary Figs. 1 and 2 and Supplementary Video 1). Defects spontaneously form due to the deposition of the filaments on the SLB, which acts as a cushion for the sedimentation of actin filaments; hence, their specific shape is due both to mechanical properties of the filaments and to the friction between the membrane and the actin. Additionally, since in this system motors are bound on a diffusive substrate, steric interactions are enforced between gliding filaments and passive ones, as previously shown^9,33,34^. Additional steric interactions between actin filaments are due to the presence of a depletant. MTs are therefore forced to glide inside the nematic material and to locally align with actin filaments (Fig. 1b and Supplementary Video 2). After stabilization of the actin nematic, we observe that the MTs, which are initially isotropic in space, start to aggregate and form long streams that span the entire sample (Fig. 1c,d). Strikingly, in these MT streams, all filaments move in the same direction (Fig. 1d and Supplementary Video 3) following the local orientation of the actin nematic (Fig. 1e). The same behaviour is observed even when varying the mechanical properties of the nematic material or the motors’ concentration, as long as filaments can move in the nematic. In the absence of an actin nematic, instead, MTs at a similar surface concentration do not form stable patterns (Supplementary Video 4) and only transiently align (Supplementary Fig. 3).

## Motion of individual filaments

**Fig. 2:**
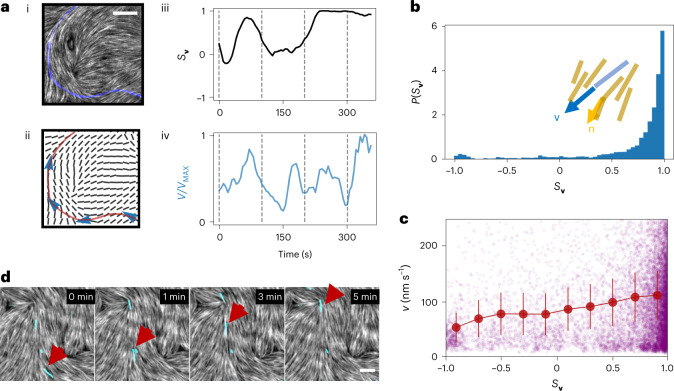
Microscopic behaviour of MTs gliding in a passive actin nematic. **a**, Example of a MT trajectory and relevant extracted parameters. Microscopy image of the nematic (grey) with the trajectory of a MT moving in it (blue) overlayed (i). Bottom left image (ii) shows the extracted nematic field (Methods) and again the trajectory, now in red. The trajectory of this MT shows how it crosses regions of different local order and that misalignment with the nematic leads to a reorientation and a drop in speed. Both the alignment with the nematic (determined by the order parameter ${{{{S}}}}_{{{\mathbf{v}}}} = 2\left( {{{{\mathbf{n}}}} \cdot {{{\mathbf{v}}}}/{{{v}}}} \right)^2 - 1$) (iii) and the speed *v* (iv) can be monitored for this trajectory over time, showing that the filament crossing defects can find itself misaligned with the nematic and thus slow down before eventually realigning. Speed is normalized by the maximum speed *v*_MAX_ during the trajectory. Dashed lines in iii and iv correspond to the positions marked by blue arrows in ii. Scale bar, 5 µm. **b**, Histogram of the probability *P*(*S*_**v**_) of observing a MT with a given value of *S*_**v**_ during its trajectory. On average, MTs are mostly aligned with the passive nematic, with *S*_**v**_ ~1. The inset illustrates the definition of **v** (in blue) and **n** (in yellow). **c**, Plot of the speed as a function of *S*_**v**_ for several MTs, sampled over the course of a video (30 minutes). The speed of the MTs depends slightly on the local alignment with the nematic *S*_**v**_, resulting in slower speed at low alignment. Purple dots are individual data points and red lines indicate the mean with standard deviation. For **b** and **c**, we tracked 400 trajectories of at least 3 minutes from three independent experiments and divided them into ten bins depending on the value of *S*_**v**_. Data are presented as mean value +/− standard deviation for each bin. **d**, Different MTs (three in this case, in cyan) end up gliding along the same trajectories. One filament (the same in all time frames) is indicated by the red arrow as a reference. Scale bar, 5 µm. Source data

To understand the microscopic behaviour leading to the formation of MT streams, we observe the system at a low MT surface density (*σ* = 0.003 MTs µm^–2^) using total internal reflection fluorescence microscopy; thus, we are able both to follow the trajectory of individual MTs inside the nematic (Fig. 2a) and to extract the local orientation **n**(**r**) of the actin filaments at any point **r** of the image (Methods). MTs are found to glide with a velocity **v** at a mean speed <*v*> ~ 100 nm s^–1^.

From the velocity, we compute the order parameter ${{{{S}}}}_{{{{\mathbf{v}}}}} =$ $2\left( {{{{\mathbf{n}}}} \cdot {{{\mathbf{v}}}}/{{{v}}}} \right)^2 - 1$, which measures the alignment between MTs and the actin nematic surrounding them. The *S*_**v**_ is expected to be 1 if MTs are aligned with the nematic field and –1 if orthogonal. Figure 2a reports an example of a MT’s trajectory, showing periods of high and low speed, dependent on the local alignment. As the MT enters an area in which it is misaligned with the nematic, it slows down and eventually realigns, demonstrating that MTs can reorient to resolve local misalignments and escape obstacles. In general, however, MTs align with the nematic director and follow its local distortions. The distribution of *S*_**v**_ is indeed strongly peaked at *S*_**v**_ ~ 1 (Fig. 2b), indicating that gliding filaments strongly align with the nematic. The speed of MTs depends on their local alignment with the nematic, with poorly aligned filaments moving more slowly (Fig. 2c). Strikingly, over time, individual MTs end up being funnelled by the nematic on the same path and in the same direction (Fig. 2d). This suggests that the nematic alignment field selects the trajectories onto which the MTs are directed due to defect-induced distortions. We then set out to determine the microscopic effect of nematic distortions on the active system by monitoring the behaviour of MTs in the presence of different distortions in the nematic, focusing on splay (high values of (**∇**·**n**)^2^, where **∇** is the nabla operator) and bend ($\left| {{{{\mathbf{n}}}}{{{\mathrm{x}}}}\boldsymbol{\nabla} {{{\mathrm{x}}}}{{{\mathbf{n}}}}} \right|^2$) and on nematic defects themselves.

## Positive defects control pattern formation

**Fig. 3:**
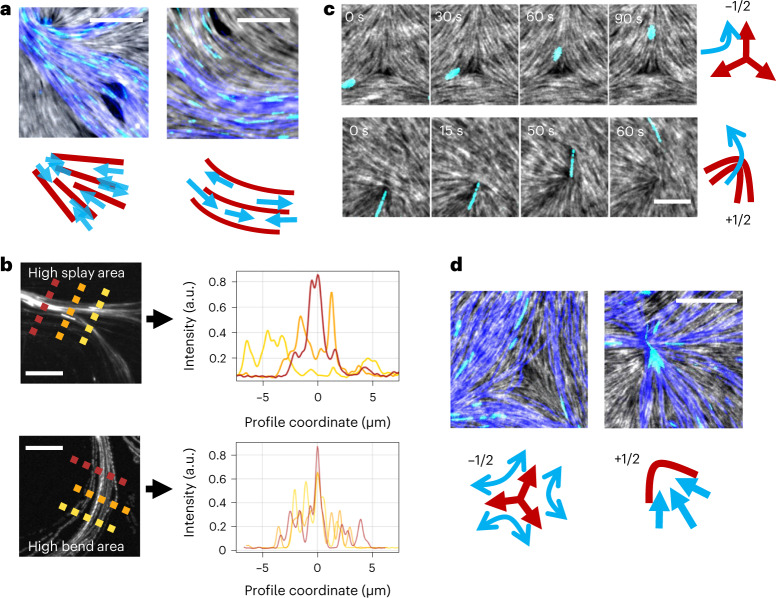
Effect of distortions in the nematic on the MT flow. **a**, In the presence of splay and bend distortions in the nematic (grey), the MT flow (blue) is affected. Insets schematize the process depicting the nematic in red and MT flow in blue. **b**, Splay (top) changes the concentration of MT streams, as shown by the intensity profile along the stream at different positions marked by different colours. The MTs’ density increases from the yellow to the red line. By contrast, bend (bottom) affects only the MTs’ orientation but not their density, and all positions show roughly the same density. In both **a** and **b**, MT density is *σ* = 0.08 MTs µm^–2^. **c**, At −1/2 defects (top), MTs are simply redirected and usually do not reach the core. At +1/2 defects (bottom), where no director is defined, MTs get directed towards the defect’s core and then are ejected out of it into the bulk of the nematic, where they realign with the local nematic director. MT density is *σ* = 0.03 MTs µm^–2^. **d**, At higher densities (MT density is *σ* = 0.08 MTs µm^–2^), the filament flow at defects behaves like individual filaments, bending at negative 1/2 defects and converging at positive ones. All scale bars, 5 µm. Insets schematize the process, depicting the nematic in red and MT flow in blue.

We observe the system at several densities (*σ* = 0.003 to 0.08 MTs µm^–2^) to extract information about both the individual and the collective behaviour of MTs. Since MTs are forced to follow the nematic orientation, splay is the source for convergence (divergence) of MT flows coming from different directions, resulting in an increase (decrease) of the density (Fig. 3a,b). In the case of pure bend instead, filaments just glide along the deformation without any change in density (Fig. 3a,b).

We next turn to the behaviour close to defects (Supplementary Video 5). Close to −1/2 defects (Fig. 3c), individual filaments simply follow the actin orientation and turn around the defect’s core before reaching it, only rarely crossing it (Supplementary Fig. 7). Hence, negative-charge defects only modify the MTs’ flow by bending its direction due to the deformations they induce. Conversely, and more strikingly, when gliding filaments enter a +1/2 nematic defect, MTs are funnelled directly to the defect’s core by the local splay, eventually escaping from the defect and realigning with the neighbouring nematic (Fig. 3c, bottom). This behaviour is the same for all observed densities (Fig. 3d), with +1/2 defects first converging and then deflecting the MTs’ flow. The actin nematic, instead, while transiently deforming when MTs glide through it, gradually recovers its initial conformation due to steric interactions with neighbouring filaments (Supplementary Fig. 4). Thus, in this system, defects are stable both in time and space yet directly affect the active flow.

## Passive defects shape the active flow

**Fig. 4:**
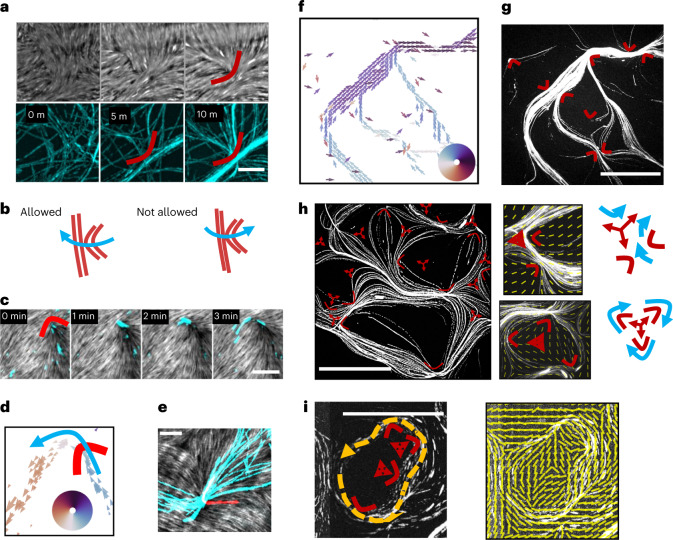
Formation of polar streams and role of defects. **a**, Positive defects (red) funnel the initially isotropic MTs into streams. The nematic is shown at the top (grey), and the MTs at the bottom (cyan). Scale bar, 5 µm. **b**, Schematic of defect-based rectification of the flow. MTs (in blue) can cross +1/2 defects (red) only from one side, thus breaking the spatial symmetry. **c**, Due to local asymmetries in the nematics, +1/2 defects break the orientational symmetry by ejecting MTs preferentially in one direction. Scale bar, 5 µm. **d**, The MT flow, extracted from videos, proves that there is a preference for MTs to move in one direction after leaving the defect. The defect’s position is marked in red. Filaments’ orientation is colour-coded according to the coloured wheel. **e**, Time projection showing MTs crossing a defect. Regardless of the way they enter, they are mostly ejected in the same direction. Over 30 minutes, 14 MTs independently go left (cyan) and only one goes right (red). Tracks of MTs not crossing the defect have been deleted for clarity (Supplementary Fig. 11). Scale bar, 5 µm. **f**, MT flow inside streams shows that they are polar. Filaments’ orientation is colour-coded according to the coloured wheel. Total time, 10 minutes. Scale bar, 50 µm. **g**, Maximum intensity time projections showing streams. They are surrounded by +1/2 defects (marked in red) that have previously funnelled MTs. **h**, Streams follow defect-induced deformations of the nematic field. Defects’ position and orientation are marked in red. Insets show close-ups of the same image (right) and schematics (far right; red, defects; blue, flow). Configurations of multiple defects shape the MT flow, for example, channelling the flow in specific directions (top) or giving rise to closed loops (bottom). Nematic field in yellow. Scale bar, 50 µm. **i**, Loops are polar and thus must enclose a total charge of +1 (*N* negative and *N* + 2 positive defects). The specific shape of the loop is due to the shape of the nematic field (yellow, on the right), which is influenced by defects inside and outside the loop. All experiments are at *σ* = 0.08 MTs µm^–2^. Scale bar, 50 µm. Source data

We find that the funnelling of MTs at defects with a positive topological charge (from now on, positive defects) is the main mechanism of pattern formation. As soon as the nematic assembles, +1/2 defects funnel initially disordered filaments into ordered structures (Fig. 4a and Supplementary Video 6).

The splay-dominated part of the defect accumulates the MTs towards its core. Furthermore, due to their conformation, positive defects can only be accessed by MTs on one side, so that they always exert a converging effect on the MTs’ density. Thus +1/2 defects break the spatial symmetry rectifying the flow^35^ (Fig. 4b). Additionally, if the nematic field right after the defect’s core is oriented at a skewed angle with the defect’s axis, most of the ejected MTs will preferentially turn in one direction, choosing the one that minimizes their rotation (Fig. 4c,d and Supplementary Video 7). We find that roughly (90 ± 10)% of the MTs independently choose the direction set by distortions in the nematic (Methods), regardless of the way they entered the +1/2 defect (Fig. 4e). Positive +1/2 defects therefore are the source of net polarity in the system, as they select a main direction, resulting in highly polar streams (Fig. 4f and Supplementary Fig. 10).

After streams have formed, the shape of the patterns is closely tied to the distribution of defects in space, as the MTs still follow the defect-induced distortions. Plotting the position and orientation of nematic defects on top of the MTs’ flow reveals how they shape the trajectories (Fig. 4g,h). After forming, streams rarely cross +1/2 defects but are surrounded by them, indicating that +1/2 defects have previously played a role in channelling the MTs into the final trajectories (Fig. 4g). Also, multiple defects arranged in specific conformations further steer the polar flow (Fig. 4h, insets). We often observe the formation of closed, chiral loops in the MTs’ trajectories (Fig. 4h, bottom inset and Fig. 4i). All observed loops have in common that they contain *N* negative defects and *N* + 2 positive ones (Supplementary Video 8). Indeed, since loops correspond to MTs ending up in the original position after a full 2π rotation, they are possible only if they enclose a total topological charge of +1, so multiple half-integer defects must play a role in forming them (Fig. 4i, left). Whether a loop will form around a total charge of +1, its precise shape and its chirality depend, on the other hand, on the shape of the nematic field in its proximity (Fig. 4i, right).

Together, these results indicate that defects play a dual role in shaping MT flow: Locally, +1/2 defects and their surroundings play a direct role by both funnelling the MTs, due to their shape, and selecting their direction, due to asymmetries in the nematic field. Globally, all defects produce deformations of the nematic material that affect the MTs’ flow even at a distance and can act in unison to create complex patterns, such as loops.

## Simulations and stream prediction

**Fig. 5:**
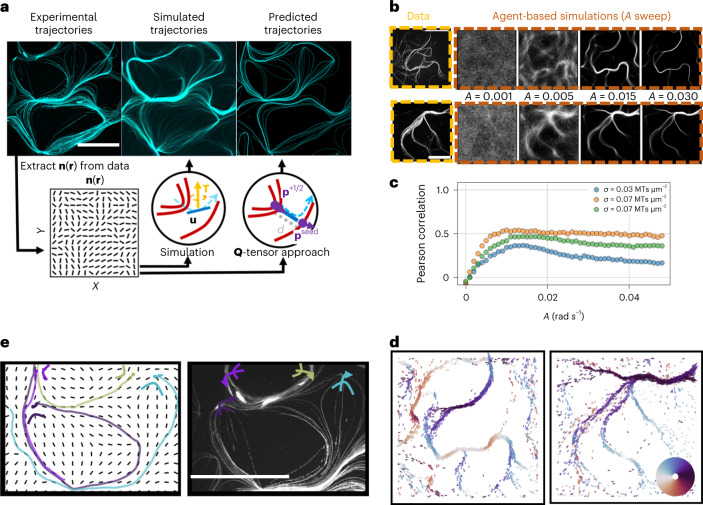
Simulations and heuristic arguments describe the experimental results well. **a**, The experimental trajectories (top left) can be faithfully reproduced by different approaches that start from a knowledge of the nematic field only. We extract the nematic field **n**(**r**) from microscopy images and then use it as input for agent-based simulations (top centre) and a *Q*-tensor-based heuristic approach to predict the streams (top right). Insets on the bottom schematize the different approaches. Both approaches reproduce the experimental trajectories. Scale bar, 50 µm. **b**, Simulations recover experimental trajectories, both at high and low MT density. Simulations rely on a local torque, indicated in the Figure by the vector **T**, acting on self-propelled particles to align them with the field. Varying its strength, the systems can either be isotropic (*A* ~ 0) or assemble into streams. Scale bar, 50 µm. **c**, As the alignment rate is increased to *A* ~ 0.01, simulations start showing very good agreement with experimental trajectories, as quantified by the Pearson correlation coefficient between experimental and simulated images (Methods). Each colour depicts a different experiment with different surface concentrations of MTs. The correlation decreases at high *A* as simulated streams become too thin. **d**, Simulations also precisely recover the polar flow of experiments (compare with Fig. 4f). The particles’ orientation is shown according to the coloured wheel, indicating that the information about the polarity is encoded in the field. **e**, To further confirm the result, the *Q*-tensor approach focuses on the role of +1/2 defects. Defects are identified in the nematic field and the morphology of their surroundings is used to identify the streamlines they generate by converging the MT flow. Each defect’s core corresponds to a colour-coded, oriented streamline (left) to be compared with experimental data (right). Only selected defects are shown. This shows that +1/2 defects directly play a role in shaping the flow. Scale bar, 50 µm.

The data reported so far suggest that the formation and morphology of polar streams are only due to the presence of defects in the nematic field and its resulting shape, combined with the fact that MTs are self-propelled. To explain this observation, we extract the field **n**(**r**) from images (Methods) and use agent-based simulations that emulate the behaviour of MTs interacting with a nematic field to predict the path the MTs will follow. We use non-interacting point-like particles that move persistently in direction **u** = (cos(*θ*), sin(*θ*)), where θ is the particle orientation, with a constant speed *v* = 0.1 μm s^–1^. Particles receive an aligning torque by the extracted nematic field **n** = (cos(*ϕ*), sin(*ϕ*)), where φ is the orientation of the nematic director. The equations of motion for a given particle at position **r** and orientation θ are

1 $$\frac{{{\mathrm{d}}{{{\mathbf{r}}}}}}{{{\mathrm{d}}t}} = v{{{\mathbf{u}}}},$$

2 $$\frac{{{\mathrm{d}}\theta }}{{{{{\mathrm{d}}t}}}} = A\;{{{\mathrm{sin}}}}\left[ {2\left( {\phi \left( {{{\mathbf{r}}}} \right) - \theta } \right)} \right] + \sqrt {\frac{{2v}}{{L_{\mathrm{p}}}}} \xi ,$$

where *t* is time and *ξ* is a Gaussian white noise with zero mean and unit variance, and its prefactor ensures a path persistence length *L*_p_ = 100 μm (Supplementary Information), guaranteeing that faster particles (higher *v*) decorrelate sooner (higher noise). The parameters *v* and *L*_p_ summarize all factors contributing to the MTs’ motion, such as the SLB diffusivity or the motors’ density^33^. The parameter *A*, measured in radians per second, is instead an alignment rate representing the strength of the coupling between particles and the nematic field and hence summarizes all factors contributing to the alignment of MTs to the actin nematic. To test the influence of alignment, we conduct parameter sweeps over the coupling strength *A*. Remarkably, as *A* is increased, we find that the simulated particles assemble into streams that closely resemble those observed in experiments (Fig. 5a–c). Simulated streams are polar and show the same orientation as experiments (Fig. 5d and Supplementary Fig. 10). Moreover, in simulations performed using randomly generated nematic fields, we observe polar streams and loops enclosing a total charge of +1 as in experiments (Supplementary Fig. 9), confirming they are general properties of defect-containing nematics. Locally apolar streams can also be observed, but they are extremely rare unless the nematic is particularly symmetric (Supplementary Figs. 8 and 11) or two oppositely polar patterns locally merge.

This numerical model agrees very well with the experimental results. Despite its simplicity, the model can also be used to quantify the streams’ polarity (Supplementary Fig. 10) and test the effect of local interparticle interactions (Supplementary Fig. 12), of mechanical properties of the nematic (Supplementary Figs. 13 and 14) including the shape and asymmetry of defects (Supplementary Fig. 11) and of persistent self-propulsion (Supplementary Fig. 15). Hence, we conclude that polar streams are an extremely general feature of self-propelled agents moving inside defect-containing nematic fields and do not depend on the microscopic details of MT motion. The only necessary assumptions for the emergence of polar streams are that particles move persistently enough, that they escape defects and that they align with the nematic field.

Thus, emerging patterns are simply a consequence of the conformation of the nematic director field **n**(**r**). Specifically, since the only points at which MTs are funnelled and deviate are +1/2 defects, they and the distortions they induce must be the fundamental elements underlying the polar pattern formation process (Fig. 4c,d and Supplementary Fig. 11). To investigate the extent to which this is the case, we also develop a polar streamline prediction approach based only on the characteristics of the nematic field in the vicinity of positive defects. The approach consists of identifying starting points for trajectories right after defects and then predicting their preferred direction depending on local distortions. These distortions are described in terms of the nematic tensor *Q* and its spatial derivatives (Supplementary Information and Fig. 5e).

First, we identify the positions **r**^+1/2^ of positive defects as local maxima of the topological charge density^36^. Due to the continuous self-propulsion of the MTs, we expect that the position of the starting points of the streamlines will be shifted with respect to the core of the defects along their axis and end in position **r**^seed^ = **r**^+1/2^ + *d* · **p**^+1/2^. Here **p**^+1/2^ signifies the axis of the defect^37^, computed from the divergence of *Q*, and *d* ~ 2 µm is a phenomenological parameter summarizing the mean distance travelled by a MT before realigning. Hence *d* is the only model-dependent parameter, being equivalent to a mixture of the parameters *v* and *A* of the numerical simulations. The position **r**^seed^ will then act as a seed for the streamlines. Finally, to choose the direction of the streamlines, we define a polarity field ${{{\mathbf{p}}}} = \boldsymbol{\nabla} \cdot {{{{Q}}}}/| \boldsymbol{\nabla} \cdot {{{{Q}}}}|$. The divergence of *Q* is indeed closely related to the mechanical properties of the passive nematic^25,32,38^, as it contains information about both splay and bend distortions and encodes the main direction along which they are amplified. MTs leaving a defect perceive this distortion and orient themselves accordingly. Specifically, we impose that the streamlines are perfectly aligned with the nematic field at position **r**^seed^ and define the preferred direction of motion as${{{\mathbf{n}}}}\left( {{{{\mathbf{r}}}}^{{{{\mathrm{seed}}}}}} \right){{{\mathrm{sign}}}}\left( { - {{{\mathbf{n}}}}\left( {{{{\mathbf{r}}}}^{{{{\mathrm{seed}}}}}} \right) \cdot \left( {{{{\mathbf{p}}}}^{{{{\mathrm{seed}}}}}} \right)} \right)$, that is, the direction that minimizes the scalar product between **n** and **p** in the seeding position. This corresponds to the fact that self-propelled particles will preferentially follow the direction that minimizes the change in their orientation. Streamlines are then evolved in the chosen direction along the nematic field from the seeding position until they reach another +1/2 defect or the edge of the image. This defect-based approach again reproduces experimental trajectories, starting uniquely from the experimentally observed nematic field **n** (Fig. 5a,e). However, it uses information only about the position and conformation of +1/2 defects, underlining their central role.

## Confined nematics lead to chiral loops

**Fig. 6:**
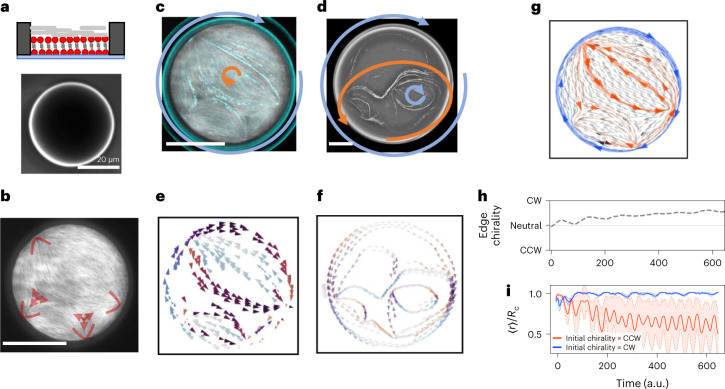
Confinement of actin nematic in a circular microwell leads to chiral loops. **a**, Schematics of the confinement (top) and top view (bottom) of a 20-µm-radius structure. **b**, Inside microwells, actin assembles into a nematic layer with multiple half-integer defects but with a total charge of +1. In this case, four +1/2 defects and two –1/2 defects are present, marked in red. **c**, Time projection of MT trajectories (cyan) in the same nematic as **b** (grey) showing a central loop with counterclockwise chirality and a clockwise edge loop. The chirality of each loop is shown by arrows. **d**, If the radius of the confining structure *R*_c_ is increased (here *R*_c_ = 50 µm), more defects are present and larger internal loops form—in this case, a smaller clockwise loop inside a larger counterclockwise loop. In both cases, an edge current is also present. The chirality of each loop is shown by arrows. **e**,**f**, Simulations reproduce the experimental patterns in **c** and **d** and also reveal the presence of an edge current. Here the time-averaged orientation of self-propelled particles is shown, colour-coded by orientation. **g**, Even if particles start at the same initial position (black arrows on the bottom), the edge current is only stable with a given chirality (blue). Particles moving in the opposite direction (orange) are ejected from the edge by distortions in the nematic and, by different paths, eventually reach a different loop inside the pattern. **h**, The chirality of particles, computed as the number of particles moving clockwise (CW) minus the number moving counterclockwise (CCW) close to the edge, quickly settles to clockwise as particles moving counterclockwise leave the edge. The time axis, shared with **i**, indicates the time in arbitrary simulation units. **i**, Depending on the initial orientation (CCW in blue, CW in orange), the distance from the centre of the pattern over time is affected. Particles initially moving counterclockwise stay at a mean distance <*r*> close to *R*_c_, whereas clockwise particles over time join the smaller inside loop (*r* < *R*_c_). Data are presented as mean +/− standard deviation among particles. Simulations were run for 50 particles with *A* = 0.03 rad s^–1^ and time is in arbitrary simulation units. All scale bars, 20 µm.

Since the formation of polar streams is a consequence of the presence of +1/2 nematic defects, a straightforward strategy to tune active patterns is confining the nematic into a circular geometry^15,39^. Then, the Poincaré–Hopf theorem^40,41^ dictates that the total nematic charge inside a disc must equal +1. Since self-assembled actin nematics feature only half-integer defects, at least two +1/2 defects must be present. Thus, because of confinement, we expect resulting polar streams to eventually form loops. The condition that the total charge equals +1 guarantees that at least one possible edge loop exists. We then perform experiments inside circular microwells carved out of a positive photoresist (Methods) with radius *R*_c_ = 20 to 130 µm (Fig. 6a) and confirm that a nematic layer with half-integer defects and a total charge of +1 assembles (Fig. 6b). We often observe the expected formation of an edge loop with a definite chirality, either clockwise or counterclockwise, but loops inside the patterns are also possible (Fig. 6c,d and Supplementary Video 9). These experiments confirm that confinement leads to the formation of loops with a clear chirality, which again is accurately captured by simulations performed using the experimental nematic field (Fig. 6e,f). Simulations also allow one to visualize trajectories close to the edges, hidden by the pattern’s autofluorescence.

Chiral edge currents arise since only one handedness leads to a stable trajectory. Defect-induced distortions indeed expel MTs gliding with the wrong handedness from the edge towards the centre; these MTs can then join an internal loop or re-enter the edge one with the right chirality. We further demonstrate this by performing simulations in which particles start at the edge with opposite directions and show that only one set of them stays along the edge (Fig. 6g,h,i). The chirality of the edge is thus determined by distortions caused by nematic defects, which this time are enclosed in the loop itself.

## Outlook

In summary, the information about the morphology and direction of polar streams is fully encoded in the orientational order of the passive nematic and its half-integer defects, and thus represents a general property of such materials that does not depend on fine details of the active system. These results are extremely general and also encompass the formation of local patterns induced by the presence of a nematic material previously observed in bacterial systems^27–30^, showing not only how patterns can result from nematic distortions, but also how global flow with a defined polarity can emerge thanks to asymmetries close to +1/2 defects. These defects indeed not only accumulate particles and rectify the flow thanks to their shape^9,29^, but also, due to local distortions, break the orientational symmetry and give rise to a globally polar flow. Moreover, multiple defects’ conformations and confinement can be used to control the system.

This system also falls into the dry active matter category^42,43^. Hence, thanks to the absence of hydrodynamic interactions, nematic defects can be fixed in space, increasing the stability of the resulting patterns and improving our ability to predict them. This suggests general strategies to drive the flow of dry active matter that do not require patterning of the whole surface^28^, as only the position and orientation of individual defects need to be controlled^30^. Moreover, in contrast with previous systems of gliding filaments^9,34,44–46^, here the emergence of global order is not a consequence of the microscopic dynamic or of filament–filament interaction, but rather of general properties of the nematic environment. Given our ability to control passive and active liquid crystals we can envision the formation of systems in which the shape of a nematic material is tuned in order to direct active patterns^19,32,47^. Additionally, in this case, the fluidity of the SLB might allow for an efficient reorientation of the MTs as the nematic is rearranged or to control the motors’ distribution in space^34^. Control of defects using confinement is also a promising approach to steer active flow. Loops around patterned +1 defects^30^ and chirality breaking in edge currents under confinement have been observed in a number of active nematic systems^15,16,48,49^. Here however, these effects arise from the positions of a few, passive, half-integer defects controlled by confinement. Altogether, these results might lead to a more targeted and energetically efficient manipulation of individual defects in nematic materials to steer active flow.

## Methods

### Buffers

The buffer used to polymerize actin (named FB25) is 2 mM Tris buffer (pH 7.5), 2 mM MgCl_2_, 0.5 mM ATP, 0.2 mM CaCl_2_ and 25 mM KCl; 1 mM DTT redox reagent was added before use.

Experiments are carried out in so-called M2B buffer: 80 mM PIPES buffer (pH 6.8), 2 mM MgCl_2_ and 1 mM egtazic acid (EGTA).

### Proteins and reagents

Biotin–kinesin and MTs stabilized with GMP-CPP (Guanosine-5'-[(α,β)-methyleno]triphosphate) were obtained from Brandeis University’s National Science Foundation Materials Research Science and Engineering Center and stored at –80 °C. Kinesin, once thawed, was kept on ice, while MTs were kept at room temperature. Actin was purified from rabbit skeletal muscle as described previously^9^. No rabbits were directly involved in the study. Monomeric actin was stored at 4 °C in the so-called G-Buffer (2 mM Tris, 0.2 mM ATP, 0.2 mM CaCl_2_, 0.2 mM DTT and 0.005% NaN_3_ at pH 8.0). Gelsolin was purified from adult bovine serum (Sigma-Aldrich). Alexa Fluor 488 phalloidin, streptavidin and Texas Red DHPE dye were purchased from Thermo Fisher. The remaining lipids (l-α-phosphatidylcholine (egg PC) and 1,2-distearoyl-sn-glycero-3-phosphoethanolamine-*N*-(biotinyl(polyethylene glycol)-2000]) (DSPE–PEG(2000)–biotin, where 2,000 is the PEG molecular weight)) were purchased from Avanti and stored in chloroform. Methylcellulose, creatine phosphate, creatine phosphokinase, catalase and pyranose oxidase were purchased from Sigma.

### Preparation of glass slides

Glass slides and coverslips (Roth) were sonicated for 20 min in a 3 M NaOH solution and then rinsed five times with double-distilled water. Afterward, they were incubated for 2 min in Piranha solution (2:1 sulfuric acid/30% hydrogen peroxide) to clean them and make them hydrophilic. Finally, they were rinsed in distilled water, in which they were stored for no more than one week. We stress that Piranha solution should be handled with care. Right before the formation of the membrane, the slides and coverslips were dried with nitrogen, and an ~50 µl observation chamber was made using a double layer of parafilm stripes as a spacer.

### Fabrication of microwells for confined nematics

To confine actin nematics, we fabricated circular microwells on the surface of glass slides. The GDS-II files of circular microwells with diameters ranging from 20 µm to 130 µm were designed using a two-dimensional CAD software (LayoutEditor). After being cleaned in the Piranha solution, the glass slides were baked at 150 °C for 30 min and spin-coated with positive photoresist (Microposit S1813G2, Kayaku Advanced Materials) at 4,000 r.p.m. Then, the photoresist was soft-baked at 90 °C, exposed at a wavelength of 365 nm using a maskless aligner (µMLA, Heidelberg Instruments) and developed by an alkaline developer (Microposit 351, Rohm and Haas). The final depth of the microwell was approximately 1.3 µm.

### Preparation of short actin filaments

A stock solution of filaments was obtained by incubating 5 µM G-actin in FB25 buffer together with 2.5 µM Alexa Fluor 488 phalloidin and 50 nM gelsolin. Filaments were polymerized for 30 minutes at room temperature and then stored on ice protected from light and used within the week. Variation of the gelsolin concentration can be used to polymerize longer or shorter filaments.

### Preparation of the SLB

SLBs were produced as in previous work^9^. Briefly, a lipid solution containing 1.25% (molar/molar, M/M) PEG(2000)–biotin, 98.75% M/M 1,2-dioleoyl-sn-glycero-3-phosphocholine (DOPC) and 0.05% M/M Texas Red DHPE was dried in a glass vial by keeping it in a vacuum chamber for at least 2 hours. The film was then hydrated in PBS buffer to a final concentration of 1 mM and then gently vortexed to dissolve lipids, sonicated for 30 minutes and extruded 20 times using Avanti’s MiniExtruder and 100 nm filers to obtain small unilamellar vesicles. The small unilamellar vesicles were protected from light and then stored on ice and used within a week. To form a bilayer, the small unilamellar vesicles are diluted to 0.33 mM, incubated for 10 minutes inside the observation chamber and then washed with at least ×10 the volume with PBS. The SLB has a diffusion coefficient of 3.4 μm^2^ s^–1^ as previously reported^9^.

### Experimental set-up

Prior to starting the experiment, a SLB was prepared and the buffer was exchanged to M2B. Biotin–kinesin was incubated 1:1 with streptavidin for 5 minutes on ice. Then, 100 nM of streptavidin–biotin–kinesin (in M2B) was incubated on the SLB for 3 minutes and then washed with M2B. This resulted in motors being bound on the SLB via the biotin–streptavidin interaction. By labelling 1 in every 10,000 streptavidin molecules with fluorescent streptavidin and counting the bright, diffusing spots on the SLB with total internal reflection fluorescence, we estimated the motors’ concentration to be 1,800 ± 500 motors μm^–2^. Kinesin on solid substrates is known to propel MTs at a speed of ~ 600 nm s^–1^ and is highly processive. Short, GMP-CPP-stabilized, Alexa-647-labelled MTs (in M2B) were then incubated for 3 minutes in the chamber at a concentration (roughly 15 µg ml^–1^) so as to obtain the desired surface density, and then washed with M2B. Finally, a mixture containing 1 µM actin filaments, 0.25% methylcellulose, 2 mM ATP and an ATP regeneration system (9 mM creatine phosphate and 18.2 units ml^–1^ creatine phosphokinase in M2B buffer) and a scavenging system (10 units ml^–1^ pyranose oxidase, 1,000 units ml^–1^ catalase and 0.66% w/w glucose) were added to the chamber, which was immediately observed using a Leica DMi8 total internal reflection fluorescence microscope with infinity scanner using a ×100, 1.47 numerical aperture, oil objective and the software LAS-X (v.3.7.4.23463). The field of view was roughly 130 µm, and images for the videos were acquired every 5 seconds. Experiments were carried out at room temperature. The MT density was estimated by counting the number of MTs inside regions of known area. The duration of each experiment was roughly 3 hours and, once formed, the actin nematic did not change visibly over the course of an experiment. Acquired data was then analysed using Fiji^50^ or custom Python3 scripts.

### Statistics and reproducibility

All experiments were performed three times per condition (low and high surface density). From each experiment, four different positions were recorded. At each position and for each experiment, the nematic field was different, but polar streams were observed in all cases. For all representative experiments shown, at least five more, different experiments exhibited the same result. Data are shown as the mean +/− standard deviation.

### Extraction of the nematic fields from images

We extracted the nematic field from the actin fluorescence channel using the method from ref. 51, encoded in a custom Python3 script. Briefly, the method assumes that the intensity gradient is perpendicular to the mean orientation of filaments. The gradient of the intensity ∇*I*(*x*, *y*) = (*I*_*x*_, *I*_*y*_) of the image is computed and the tangent vector to it **t** = (*I*_*y*_, −*I*_*x*_) := (*t*_*x*_, *t*_*y*_) is extracted and normalized. The image is then divided in boxes of length *L* × *L* = 1.3 µm × 1.3 µm spaced by 1 pixel (~0.065 µm), and inside each box the tensor *T*, analogous to the nematic tensor, is computed, with components *T*_11_ = <*t*_*x*_ *t*_*x*_>, *T*_12_ = <*t*_*x*_ *t*_*y*_>, *T*_21_ = *T*_12_ and *T*_22_ = <*t*_*y*_ *t*_*y*_>. The eigenvector of this tensor corresponds to the local nematic director at the point at the centre of the box.

### Tracking of MTs and MTs’ optical flow

Tracking was carried out using a custom Python script. Images containing active filaments are binarized with a threshold so that only MTs are selected. Their contours are identified using Python’s library OpenCV. From the contours, the position of the centre of mass (*x*_*i*_, *y*_*i*_), the alignment *Ɵ*_*i*_ and the length *L*_*i*_ of each filament *i* can be extracted. *L*_*i*_ and *Ɵ*_*i*_ are extracted by fitting the contour with an ellipse. Only contours with an aspect ratio greater than two are analysed. Trajectories are reconstructed by joining together the centres of two MTs *i* and *j* in two consecutive frames if (1) *i* and *j* are closer than 5 µm; (2) *i* is the closest contour to *j* and *j* is the closest contour to *i*; and (3) the change in area and length of the two contours is smaller than 20%. Contours that are matched are removed from further matching. From the trajectories and the contours, both the speed (computed as the distance travelled in consecutive time frames) and the alignment of contours with the nematic over time can be easily extracted, as described in the following.

The MT flow was computed from microscopy videos using Python’s cv2 library and the optical flow function. Briefly, the intensity at each pixel is correlated with that of all pixels at the following frame in a box of size 3.6 µm to find the best match and hence the flow. The local velocity is then averaged over time. The normalized velocity is then used to colour code the MT orientation. The maximum intensity time projection of the images is used to create a mask so that the flow is computed only where enough MTs are present in the image over time.

### Counting filaments leaving defects

We selected individual +1/2 defects and counted the number *n*_1_ and *n*_2_ of particles going in either of the two directions with respect to the defect’s axis. We considered only MT densities low enough that we could count individual filaments and such that MTs rarely hit each other. We selected videos with at least ten filament counts. Only filaments that independently choose their direction were considered; filaments undergoing collisions were ignored. From this, we computed the percentage of motors going in one direction as max(*n*_1_, *n*_2_)/(*n*_2_ + *n*_1_), obtaining ~(0.9 ± 0.1) as the mean +/– standard deviation (nine defects, for a total of 125 filaments counted).

### Computing the order parameters

The *S*_**v**_ was computed from the (normalized) vectors **n** of the nematic field and the velocity **v** obtained from the tracking data, thus computing the scalar product ${{{{S}}}}_{{{\mathbf{v}}}} = 2\left( {{{{\mathbf{n}}}} \cdot {{{\mathbf{v}}}}/|{{{\mathbf{v}}}}|} \right)^2 - 1$.

### Computing the Pearson coefficient

Simulated images were produced by creating 2,048 × 2,048 images, sized the same as the experimental images, with each pixel value counting the number of times a MT crossed that pixel during a simulation. Both the experimental time projections and the simulated images were then smoothed, averaged down to 256 × 256 to reduce noise and compared using the Pearson correlation coefficient. The coefficient is computed as $r = \frac{{\mathop {\sum}\nolimits_{\left( {x,\;y} \right)} {\left( {I_1\left( {x,\;y} \right) - I_1^{\mathrm{m}}} \right)\left( {I_2\left( {x,\;y} \right) - I_2^{\mathrm{m}}} \right)} }}{{\sigma _1\sigma _2}}$ where *I*_*i*_(*x*, *y*) is the intensity of image *i* at position (*x*, *y*), $I_i^{\mathrm{m}}$ the mean intensity and *σ*_*i*_ the standard deviation of the intensity over the whole image *i*.

### Reporting summary

Further information on research design is available in the Nature Portfolio Reporting Summary linked to this article.

## Online content

Any methods, additional references, Nature Portfolio reporting summaries, source data, extended data, supplementary information, acknowledgements, peer review information; details of author contributions and competing interests; and statements of data and code availability are available at 10.1038/s41563-022-01432-w.

## Supplementary information

Supplementary Information Supplementary Figs. 1–15, video legends, text and references.

Reporting Summary

Supplementary Video 1 Assembly of an actin nematic on top of a SLB. Time is in minutes. Scale bar, 100 µm.

Supplementary Video 2 Gliding MTs move inside the actin nematic and locally align to it. Time is in minutes. Scale bars, 50 µm.

Supplementary Video 3 Polar streams formed by MTs. Time is in minutes. Scale bars, 50 µm.

Supplementary Video 4 In the absence of an actin nematic, MTs do not assemble into any pattern. Time is in minutes. Scale bars, 50 µm.

Supplementary Video 5 Behaviour of individual MTs close to nematic defects. Time is in minutes. Scale bars, 5 µm.

Supplementary Video 6 As the actin nematic sediments, initially isotropic MTs start being funnelled into specific trajectories. The video is followed by a maximum intensity time projection showing accumulation. Time is in minutes. Scale bar, 50 µm.

Supplementary Video 7 Defects of positive charge (+1/2) funnel MTs and eject them preferentially in one direction. Time is in minutes. Scale bar, 5 µm.

Supplementary Video 8 MTs can assemble into chiral loops enclosing a total nematic charge of +1. Time is in minutes. Scale bar, 50 µm.

Supplementary Video 9 When confined in a circular microwell, MTs form chiral loops, comprising an edge current and inside loops. Time is in minutes. Scale bar, 20 µm.

## Data Availability

Data generated or analysed during this study are included in the Supplementary Information. Raw data necessary to reproduce the results, including the source files of videos and images, are available on a Zenodo repository (10.5281/zenodo.7071792). Further data are available from the corresponding author upon request. Source data are provided with this paper.

## Source data

Source Data Fig. 2 Source data for the plots in Fig. 2.
Source Data Fig. 4 Nine different defects analysed (including the one in Fig. 4e), showing the asymmetry in the direction at which the filaments are ejected.

## Code availability

A minimal script to extract the nematic field from images and further example code are available on Zenodo (10.5281/zenodo.7071792), together with scripts to reproduce the analysis performed.
