# Supplementary material for: Polarity and chirality control of an active fluid by passive nematic defects
Source: Nat Mater. 2022 Dec 30;22(2):260–8. doi: 10.1038/s41563-022-01432-w (PMC9894751; doi:10.1038/s41563-022-01432-w)
Supplement: Supplementary file 1 — Supplementary Figs. 1–15, video legends, text and references. [file 41563_2022_1432_MOESM1_ESM.pdf]

nature materials

Article https://doi.org/10.1038/s41563-022-01432-w

Polarity and chirality control of an active fluid by passive nematic defects

In the format provided by the authors and unedited

Supplementary Videos Legends

Supplementary Video 1: Assembly of an actin nematic on top of a supported lipid bilayer. Time is in minutes. Scale bar is 100 µm.

Supplementary Video 2: Gliding microtubules move inside the actin nematic and locally align to it. Time is in minutes. Time is in minutes. Scale bars are 50 µm.

Supplementary Video 3: Polar streams formed by microtubules. Time is in minutes. Scale bars are 50 µm.

Supplementary Video 4: In the absence of an actin nematic, microtubules do not assemble into any pattern. Time is in minutes. Scale bars are 50 µm.

Supplementary Video 5: Behavior of individual microtubules close to nematic defects. Time is in minutes. Scale bars are 5 µm.

Supplementary Video 6: As the actin nematic sediments, initially isotropic microtubules start being funneled into specific trajectories. The movie is followed by a max intensity time projection showing accumulation. Time is in minutes. Scale bar is 50 µm.

Supplementary Video 7: Defects of positive charge (+1/2) funnel microtubules and eject them preferentially in one direction. Time is in minutes. Scale bar is 5 µm.

Supplementary Video 8: Microtubules can assemble into chiral loops enclosing a total nematic charge of +1. Time is in minutes. Scale bar is 50 µm.

Supplementary Video 9: When confined in a circular microwell, microtubules form chiral loops, comprising an edge current and inside loops. Time is in minutes. Scale bar is 20 µm.

I. SELF-PROPELLED AGENT-BASED SIMULATIONS

We simulate microtubules gliding in a nematic field using an overdamped Langevin equation.

In our agent-based model, we consider spherical self-propelled particles ($N = 50$) of diameter $\delta = 1\ \mu m$. The particles move with velocity $v = 0.1\mu m/s$ in the plane and their positions can be described as:

$$\frac{\mathrm{d}\mathbf{r}^i}{\mathrm{d}t} = v\mathbf{u}^i, \tag{S1}$$

where $\mathbf{u}^i = (\cos(\theta^i), \sin(\theta^i))$ is the orientation of particle i.

The individual particle has a nematic alignment with an external field $\mathbf{n}(\mathbf{r}) = (\cos(\varphi(\mathbf{r})), \sin(\varphi(\mathbf{r})))$, with the alignment rate A, and with neighbors within the interaction radius $\delta = 1\ \mu m$, with the alignment rate s:

$$\frac{\mathrm{d}\theta^i}{\mathrm{d}t} = A\sin\left(2[\varphi(\mathbf{r}^i) - \theta^i]\right) + s\sum_{j\neq i, r_{ij}<\delta} \sin\left(2[\theta^j - \theta^i]\right) + \sqrt{\frac{2v}{L_p}}\xi\,. \tag{S2}$$

where ξ is random Gaussian white noise with zero mean and unit variance with a pre-fractor ensuring a path persistence length of $L_p = 100\ \mu m$. The factor of two inside the sine terms implements nematic alignment between particles, i.e. it is the same under the transformations $\phi \to \phi + \pi$ or $\theta^i \to \theta^i + \pi$.

A represents the alignment rate between the nematic field and the particles, whereas s that of interaction between particles. If $s = 0$, as used in the main text, particles are effectively point-like and independent. The alignment between particles and the nematic field can be summarized by the order parameter

$$S_u = 2\left(\mathbf{u}^i \cdot \mathbf{n}(\mathbf{r^i})\right)^2 - 1 \tag{S3}$$

If not stated otherwise, the simulation was configured as follows: The nematic background fields had a resolution of 2008×2008, with a pixel edge-length of $64.5nm$. The density of particles was set to be $0.03/\mu m^2$. Simulations were initiated with random initial conditions (i.e. particles initially placed at random positions and with a random orientation). To cut off initial transients, we let the simulation proceed for 2000 s before starting to obtain measures. These were subsequently recorded in 800 frames, equally spaced over a time-window of 8000 s.

Measures in the experiments were taken with a microscopic field of view much smaller than the whole experimental system, hence the nematic field on opposing sides as well as particles exiting resp. entering the observed area of the system are not correlated (see e.g. Fig. 2A). To account for this fact in the simulations, we used "grand canonical" boundary conditions when simulating an experimentally obtained field. Here, when particles cross a boundary, they are put at a random position along one randomly chosen side of the simulation box with a randomly chosen orientation. Otherwise we used periodic boundaries.

In simulations from nematic fields extracted from confined nematics, the extracted field is surrounded by an additional circular field to mimic the tangential boundary condition and avoid that particles escape confinement.

II. SIMULATED FIELDS ALSO CONFIRM THE RESULTS

To verify the existence of polar streams in the case of randomly oriented but realistic defects we generate artificial nematic patterns by integrating the equation for relaxational dynamics of passive nematics. Particularly we use the simplest form of Landau-de Genne's free energy F_{LdG} with one-elastic-constant approximation:

$$\partial_t Q_{ij} = -\frac{1}{\gamma}\left(\frac{\delta F_{LdG}}{\delta Q_{ij}}\right)^{ST} = \left[\alpha - \beta Q_{kl}Q_{kl}\right]Q_{ij} + L_1\Delta Q_{ij}, \tag{S4}$$

where γ is rotational viscosity (which is eliminated by the time rescaling), and symbol $(...)^{ST}$ denotes symmetric and traceless part of the tensor. Hereafter, we will always make use of the Einstein notation for summation over repeated indices. Elasticity coefficient L_1 is Landau-De Gennes's coefficient in one constant approximation [1]; α and β are standard Ginzburg-Landau coefficients selected in a such way to set the equilibrium value of scalar order parameter $Q_0 = 1$. The equation is solved using arbitrary dimensionless units and then, if necessary, the dimensions of the simulated nematic are converted to microns using roughly 1 pixel = 1 μm so that it resembled the size of the experimental fields. We stop the time evolution of the field before the system reaches an equilibrium homogeneous state and use the output as the pre-imposed director field for our agent-based simulation. In this case, particles also form polar streams "touching" the +1/2 defects.

To address the formation of polar streams, we consider the behavior of particles on a two-dimensional square periodic domain of the size L with synthetically generated nematic fields containing simple configurations of topological defects. We firstly consider two pairs of symmetrically located $\pm 1/2$ defects, and introduce the following director field: $\mathbf{n} = (\cos(\phi), \sin(\phi))$, where $\phi = \sum_i k_i \tan^{-1}((y - y_i^0)/(x - x_i^0)) + \phi_0$. Here k_i and (x_i^0, y_i^0) define topological charge and the core's position of the i_{th} defect, and ϕ_0 is initial angle. Particularly,

$$k_1 = -1/2, (x_1^0, y_1^0) = (-l, l) + (x_c, y_c), \tag{S5}$$

$$k_2 = 1/2, (x_2^0, y_2^0) = (l, -l) + (x_c, y_c), \tag{S6}$$

$$k_3 = 1/2, (x_3^0, y_3^0) = (-l, l) + (x_c, y_c), \tag{S7}$$

$$k_4 = -1/2, (x_4^0, y_4^0) = (-l, -l) + (x_c, y_c), \tag{S8}$$

where the $(x_c, y_c) = (L/2, L/2)$ is the center of the domain, and $l \approx L/4$.

Running the simulations on the introduced director field, we observe that particles form nematic lane, which passes in the vicinity of positive topological defects, perpendicular to their axis (**Supplementary Figure 8**A). That goes in line with the fact that topological defects correspond to the maximal distortion of the nematic field: splay deformation leads to the convergence of the nematic streamlines in $+1/2$ disclinations (and divergence in $-1/2$ ones) [2]. Thus, positively charged defects play the role of natural attractors, although the lane is shifted from the defect core by a small distance along the defect's axis.

However, if one introduces small perturbation $\mathbf{n}_\delta = \delta(0, \cos(2\pi x/L))$, particles moving in a distorted field $\mathbf{n}_d = (\mathbf{n} + \mathbf{n}_\delta)/|(\mathbf{n} + \mathbf{n}_\delta)|$ form a polar stream (**Supplementary Figure 8**C). This example illustrates how a deviation of the field around the defect from a perfectly symmetric one can lead to a symmetry breaking.

The possibility of running simulated fields also allows us to confirm that what we observe is a general characteristics of random nematic materials. Remarkably, for example, for some configurations of defects we even observe the polar rings discussed in the main text (**Supplementary Figure 9**B).

The agent based model also allows for a more direct quantification of the polarity of streams in general. We started from the field extracted from experiments shown in the main text as Figure 4F, 4G and 5D, run simulations on it at different values of the alignment strength A and then computed the degree of local polarity in the systems as follows. First, the image is divided in a 100×100 grid. Inside each "box" m of the grid, the orientation $\mathbf{u}$ of each particle crossing the grid during the simulation is recorded. We then calculate, for each box, the absolute value of the total polarity $v_m = |\sum^{N_m} \mathbf{u}^i|$, where the index i runs over all N_m particles that crossed cell m. With this we define the total amount of local polar order in the system

$$\mathbf{P} = \frac{1}{\sum_m N_m} \sum_m v_m \tag{S9}$$

(where only cells with $N_m > 30$ were considered). The result (**Supplementary Figure 10**) clearly shows that as A is increased, the polarity in the system strongly increases.

As a complementary way to demonstrate the degree of polarity, we additionally checked for each particle crossing a box m during the simulation, whether its orientation is within $60°$ of the mean orientation (averaged over all particles N_m crossing box m; again only cells with $N_m > 30$ were considered). In **Supplementary Figure 10** we plotted the fraction of particles moving within this threshold against the total number of particles analysed.

The result clearly shows that as A is increased more than 90% of the particles move coherently inside the stream.

III. HEURISTIC STREAMLINE PREDICTION APPROACH

Armed with the knowledge that small perturbations around $+1/2$ defects are at the base of polar flow, we can formulate our heuristic rule predicting polar streamlines' location. It consists of (i) identifying a starting point of the potential stream and (ii) determination of its direction. We first identify the positions $\mathbf{r}_i^{+1/2}$ of positive defects as local maxima of the topological charge density q [3, 4] defined as:

$$q = \frac{1}{4\pi} \left(\partial_x Q_{xi} \partial_y Q_{yi} - \partial_x Q_{yi} \partial_y Q_{xi} \right), \tag{S10}$$

where Q_{ij} is a tracelesss and symmetric tensor describing an average alignment of filaments disregarding their orientation, defined as $Q_{ij} = \langle n_j n_j - \delta_{ij}/2 \rangle$ with δ_{ij} denoting Kronecker delta and repeated indices indicate summation.

From the symmetry grounds, the only source of the polarity coming from the actin layer is the divergence of the nematic field. We define the local actin-induced polarity (or just "polarity" hereafter) as $p_i = -\partial_j Q_{ij}/|\partial_j Q_{ij}|$.

Accordingly, the axis of $+1/2$ defect can be defined as averaged p_i at the defect core: $a_i = p_i^{+1/2}$ [5]. Due to the continuing self-propulsion of the particles, we expect that position of the streamline starting point (seed) is shifted in respect to the defect core: $r_i^{+1/2} + \mathrm{d}\, a_i$ where d is a small phenomenological parameter depending on the alignment strength A. It can be explained both by the particles overshooting and curvature-induced flux [6, 7].

The parameter d would depend on both the speed v and the alignment rate A. The choice of $d \approx 2.5\ \mu m$ made in the main text is arbitrary and is based on the fact that microtubules align right after the defect at an offset comparable to their length. In more general terms, $d = 2.5\ \mu m$ would correspond to the distance travelled by a particle moving at $v = 100\ nm/s$ and aligning at a rate $A = 0.02$ rad/s (roughly the value at which particles start aligning strongly to the nematic in our conditions) before de-correlating its initial direction, due to the effect of the nematic, by an angle of $\approx \pi/4$. However, we found that the precise value of d does not matter much.

Finally, preferred direction of motion can be defined as $n_i \mathrm{sign}(-n_k \partial_j Q_{kj})$ at the stream line's starting point. As one can see, if topological defect is perfectly symmetric actin-induced polarity is normal to the local director ($n_k \partial_j Q_{kj} = 0$), consequently no symmetry breaking can be observed and the stream remains nematic. However, the symmetry breaks down when director field departs from the ideal profile. Any imperfections (e.g. inherent "twist" [8], or the influence of adjacent defect [5, 9]) can result in polar streams.

Our heuristic rule is pictorially illustrated in **Supplementary Figure 8**E; blue arrows correspond to the polarity field (p_i), grey segments are for the nematic alignment. When a particle moves from left to right being funneled by wedge-shape form of $+1/2$ defect, it reaches defect core region (green dot) and overshoots by distance d keep moving along the defect axis ($p_i^{+1/2}$). At this point (blue dot), the fate of particle is determined by the projection of local polarity (p_i^{seed}) on the nematic director. A smaller angle between polarity and $\pm n_i$ corresponds to a higher probability for particles to turn to the corresponding direction (green arrow). The introduced heuristics being applied to two mentioned director fields (**Supplementary Figure 8** A, C) reproduce the localization of particles and their orientation with a high level of fidelity (**Supplementary Figure 8** B, D, respectively).

It is worth mentioning, that the divergence of the Q-tensor contains both splay and bend deformations: $\partial_j Q_{ij} \propto n_i \partial_j n_j + (n_j \partial_j) n_i$. Splay deformation itself can concentrate particles and lead to the formation of the polar streams [10] (see the results for $\mathbf{n} = (\cos(\pi y/L), -\sin(\pi y/L))$ in **Supplementary Figure 9**A). Bend deformation, on contrary, can lead to the nematic stream (not shown). However, in the real passive nematics, these two types of distortion are typically localized around the defects, which is often energetically beneficial.

IV. ADDITIONAL RESULTS FROM THE NUMERICAL MODEL

The agent-based model introduced in section I allows to further verify how different properties of the particles' motion and of the nematic material influence the formation of polar streams.

A. Effect of local asymmetries in the shape of defects

To further confirm our argument that polarity emerges due to asymmetries in the nematic field in proximity of $+1/2$ defects, we start from a nematic field $\mathbf{n}$ containing a perfectly symmetrical $+1/2$ defect and add gradual distortions in the form of $\mathbf{n} \to \mathbf{n} + \mathbf{n}_\delta = \mathbf{n} + \delta(0, \cos(2\pi x/L))$, where δ is the strength of the distortion. As the defect becomes more and more asymmetric, filaments start leaving it preferentially in one direction, confirming that asymmetries favor the formation of polar streams (**Supplementary Figure 11**).

B. Effect of interaction

As in experiments it is often witnessed that microtubules interact, particularly when converged by a $+1/2$ defect, we tested the influence of interaction between agents. The result, depicted in **Supplementary Figure 12**, shows that even when the interaction parameter s (Eq. S2) is increased, no significant effect is observed in the ability of particles to align with the nematic field (**Supplementary Figure 12**).

C. Effect of the nematic's elasticity and of defect's shape

To investigate the effect of the nematics' elasticity, we simulate artificial fields with different values of the elastic constant L_1. In all cases, we observe the formation of polar lanes, indicating that the presence of defects alone is

sufficient for the sorting of the polarity. Yet, as the effect of L_1 is tuning the shape of the deformations, we do observe that as L_1 is increased, the density of defects and the total amount of deformation lowers, leading to wider lanes (nevertheless polarity is conserved). This suggests that the material properties of the nematic can be tuned to shape the patterns, but that the polarity is a general consequence of the presence of multiple random +1/2 defects (**Supplementary Figure 13**).

In order to quantify the degree of polarity we divided the systems into grids with a box edge-length of $3\mu m$ and calculated $\mathbf{P}$ (Eq. S9).

D. Effect of defect's shape

Changing the nematic mechanical properties also can have an influence on the shape of defects. In particular, it the splay-to-bend elastic constant ratio is varied, defects can become more "pointy" (if splay is favored) or round (vice-versa). To test this, we start from the two-dimensional version of equations derived in [1] and compute fields with defects of different shapes. Particularly, to break the degeneracy between splay and bend elastic constants we introduce additional term to elastic part of Landau-De Gennes free energy (see Eq. S4):

$$f_{el} = \frac{1}{2} L_1 \left(\partial_k Q_{ij}\right)\left(\partial_k Q_{ij}\right) + \frac{1}{2} L_3 Q_{ij} \partial_i Q_{kl} \partial_j Q_{kl}. \tag{S11}$$

$$L_1 = \frac{1}{2Q_0^2}\left[K_{11} + \frac{1}{2}\left(K_{33} - K_{11}\right)\right], \tag{S12}$$

$$L_3 = \frac{1}{2Q_0^3}\left[K_{33} - K_{11}\right], \tag{S13}$$

where Q_0 is the equilibrium value of scalar order parameter (which is set to unity by choosing the α and β terms of Eq. S4), K_{11} and K_{33} are splay and bend Frank-Oseen elastic constants, respectively [1, 11]. The ratio between the corresponding Landau-De Gennes coefficients, defining the imbalance between splay and bend, reads: $z = L_3/L_1 = (\epsilon - 1)/(2(\epsilon + 1))$, where $\epsilon = K_{33}/K_{11}$.

Again, no effect is observed in the polarity but only in the shape of the polar streams (**Supplementary Figure 14**).

E. Effect of speed reversal

Microtubules are polar particles that move persistently in one direction. Other active particles (e.g. bacteria) instead can spontaneously change their direction as they move or swim. What is the effect of the nematic field in this case? To test this, we started from the assumption that particles *do* align with the nematic field (i.e., the parameter A is high enough). In this sense, the persistence length L_p plays a minor role as the path persistence length is rather fixed by the nematic field since the orientational noise of particles is not strong enough to overcome the alignment A. Hence the only thing that a particle can do except aligning is to reverse their direction of motion. This amounts to particles not being polar anymore but rather nematic. We then introduced a reversal rate λ in our model. Roughly every $1/\lambda$ seconds, the particles can reverse their speed, i.e. their orientation flips by an angle π ($\theta \to \theta + \pi$). This introduces a new persistence length $L_r = v/\lambda$ over which particles move persistently, before reversing their speed.

The results, summarized in **Supplementary Figure 15**, are as follows (at fixed $A = 0.02$ rad/s in the example here shown): for very high λ, particles on average do not move and hence barely form any patterns in the given simulation times and no net polarity is present. Notice however that even in this case, every time a particle crosses a +1/2 defect it will still align with the field and hence be rectified (according to the mechanism sketched in Fig. 4B) and hence particles still accumulate along the trajectories where patterns would form at $\lambda = 0$! (Simulations over very long time scales do indeed show pattern formation in this case as well, since particles "explore" the nematic by diffusion). For intermediate λ, patterns form but are weakly polar as inside the patterns particles can still "flip". Strikingly, the shape of the patterns is similar to the ones observed for $\lambda = 0$, since as long as particles can move long enough to cross defects the rectification effect due to +1/2 defects is still at play (but not the polarity sorting mechanism due to asymmetry). At low λ, patterns are polar. We show this by computing both the mean polar order (1 if patterns are completely polar, lower otherwise) and the similarity of patterns at a given λ with the expected ones at $\lambda = 0$ (again, using Pearson's correlation coefficient). Clearly, the similarity between patterns is high for intermediate λ even when the polarity is low. These results further indicate that, for polar patterns to arise, particles need to persistently move in one direction for a long enough time, set by the typical length of the nematic field. Hence polar streams are a consequence of persistent self propulsion in the presence of defects and asymmetries. This also

hints that nematic particles (that change their speed often but not too often) would accumulate in patterns but their symmetry would be nematic, i.e. patterns in which particles move in both directions.

Supplementary Figures

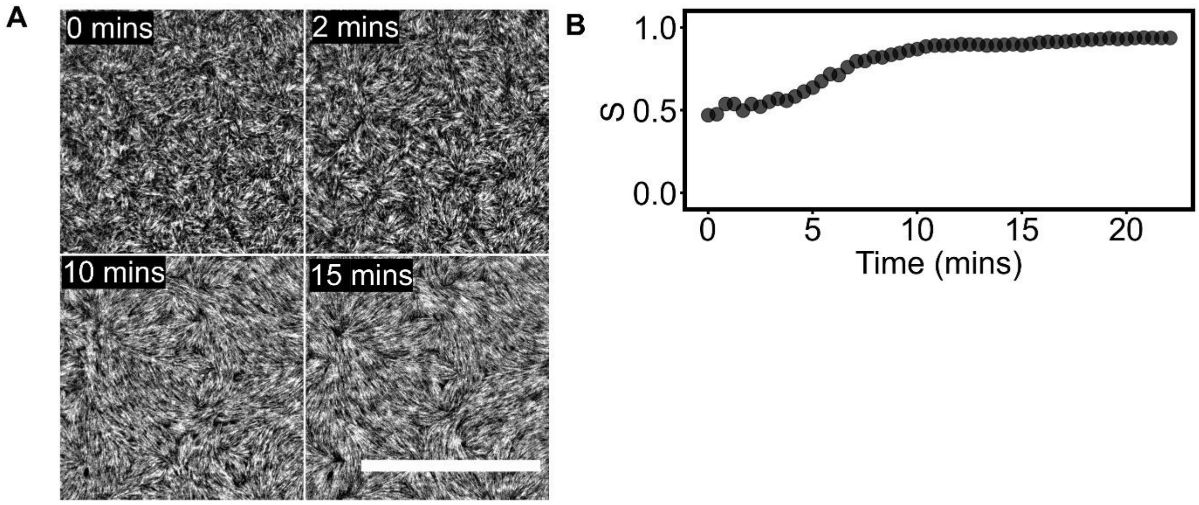

Supplementary Figure 1. *An actin nematic composed of small stabilized filaments assembles quickly over the course of 10 minutes (A), as shown by the increase in the nematic order parameter S over time (B). Scale bar is 50 µm.*

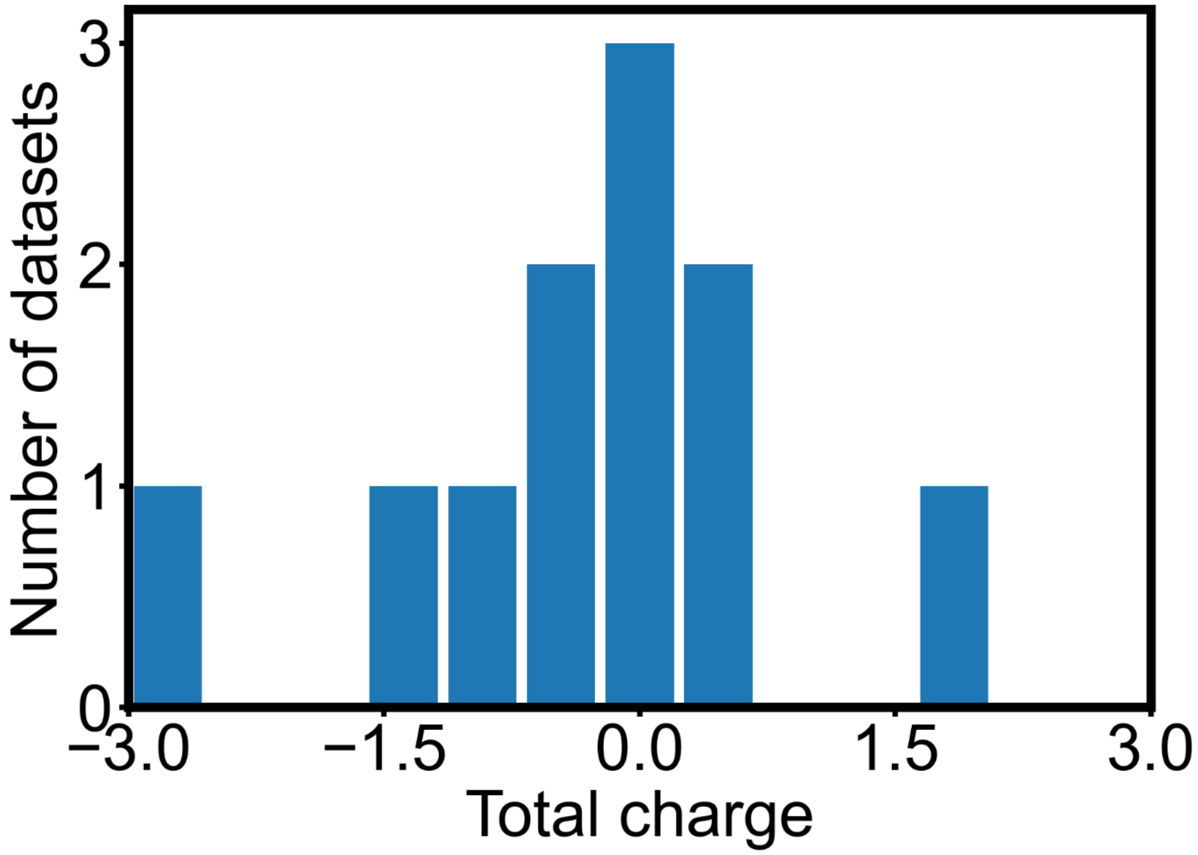

Supplementary Figure 2. *The total charge of the observed nematic field is close to 0 in all experiments, with deviation due to the limited area of the field of view.*

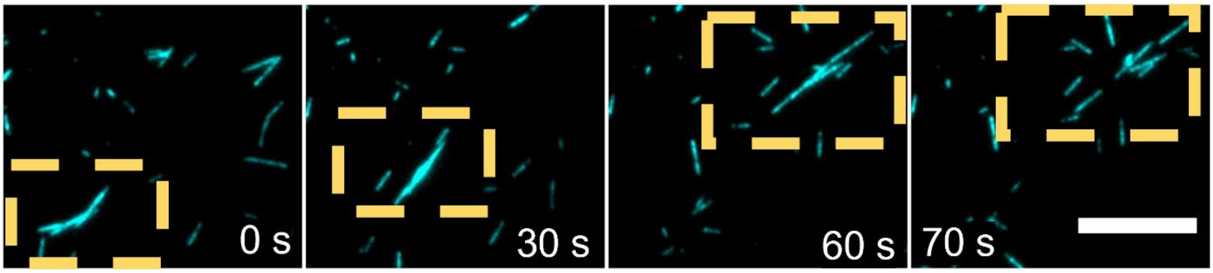

Supplementary Figure 3. *In the absence of a passive nematic, even at σ≈0.07 MTs/µm² MTs do not form stable lanes but only transient clusters that merge and disaggregate over time. Scale bar is 10 µm.*

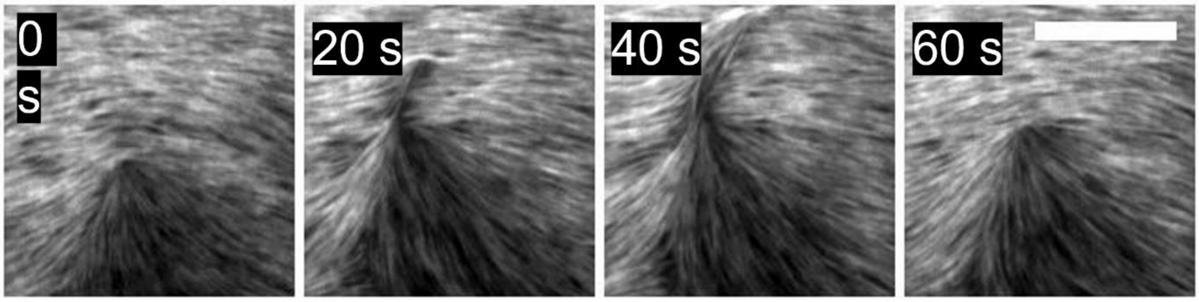

Supplementary Figure 4. Microtubules escaping a +1/2 defect locally deform the actin nematic. As soon as they escape, the nematic reverts almost completely to its original shape due to steric interactions between the actin filaments. This effect, considering that the numerical model predicts the right patterns without taking it into account, is assumed to be negligible. Scale bar is 10 µm.

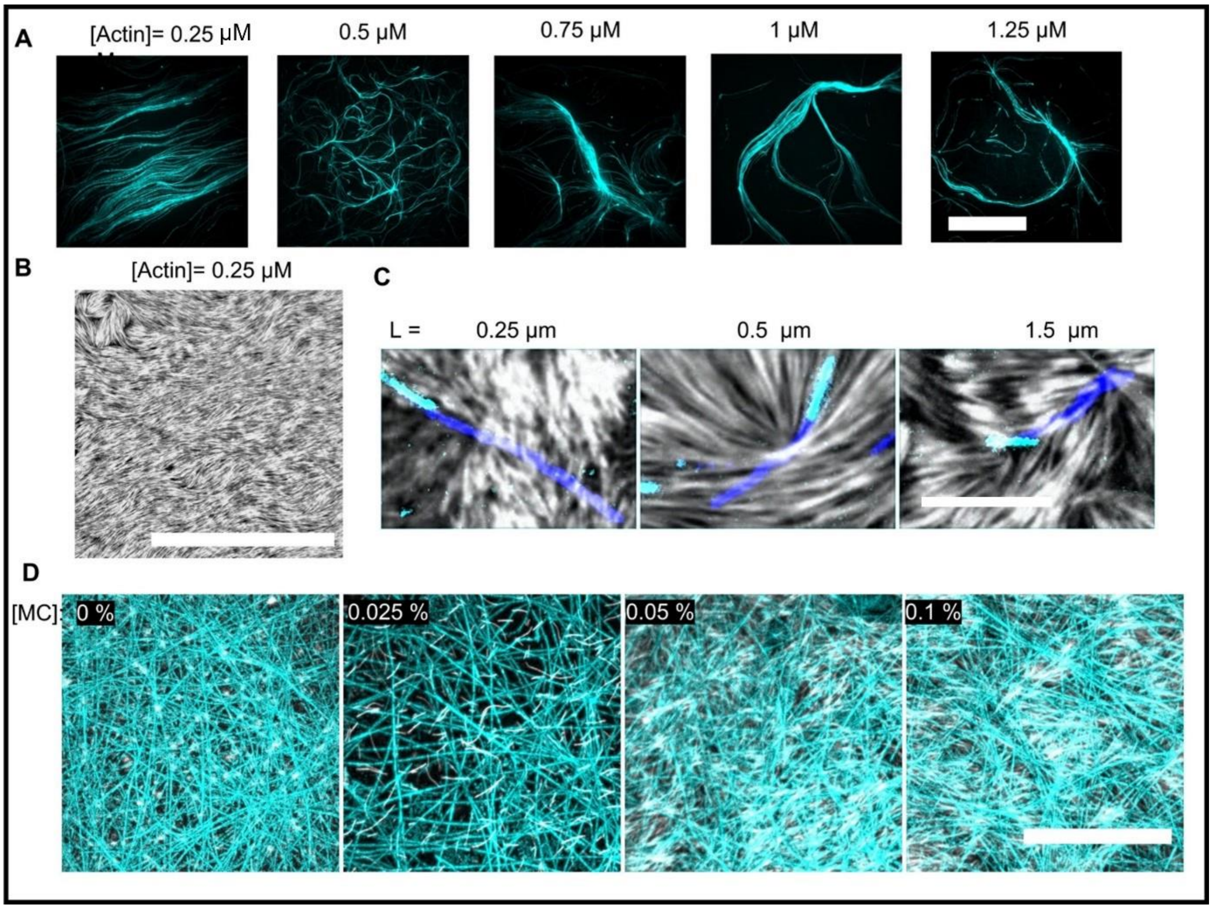

Supplementary Figure 5. *A) Changing the amount of actin has only a minimal effect on the formation of lanes, as long as a nematic forms. Polar patterns always form. B) The nematic field corresponding to the trajectories shown above for 0.25 µM of actin. At this very low actin density, the nematic can be deformed (in this case, aligned) by the microtubules' streams. C) Changing the filament lengths using higher concentrations of gelsolin affects the behavior at defects. Time projections of MTs (cyan) escaping defects in nematics composed of actin filaments of different length (gray). As the filaments get longer, MTs align faster with the nematic after being ejected by the defect's core. Filaments are polymerized, from left to right, with 100, 75 and 25 nM of gelsolin. Scale bar is 5 µm. D) Tuning the methylcellulose (MC) concentration, so that actin (in grey) sediments but the nematic does not form, shows that, unless a full, stable nematic forms no polar lanes arise. MTs' trajectories are in cyan, actin filaments are shown in grayscale. At very low MC (0%-0.025%), only a few actin filaments sediment transiently and trajectories of MTs are straight and do not form any pattern because of the absence of a nematic. At intermediate MC (0.05-0.1%) even if a nematic forms, at this concentration of depletant it is not stable but changes shape over time as filaments can escape fluctuating in the third dimension. Hence again no pattern forms. Patterns are stable only for a concentration of depletant above 0.2-0.25%. Scale bars are 50 µm except in defects close-up.*

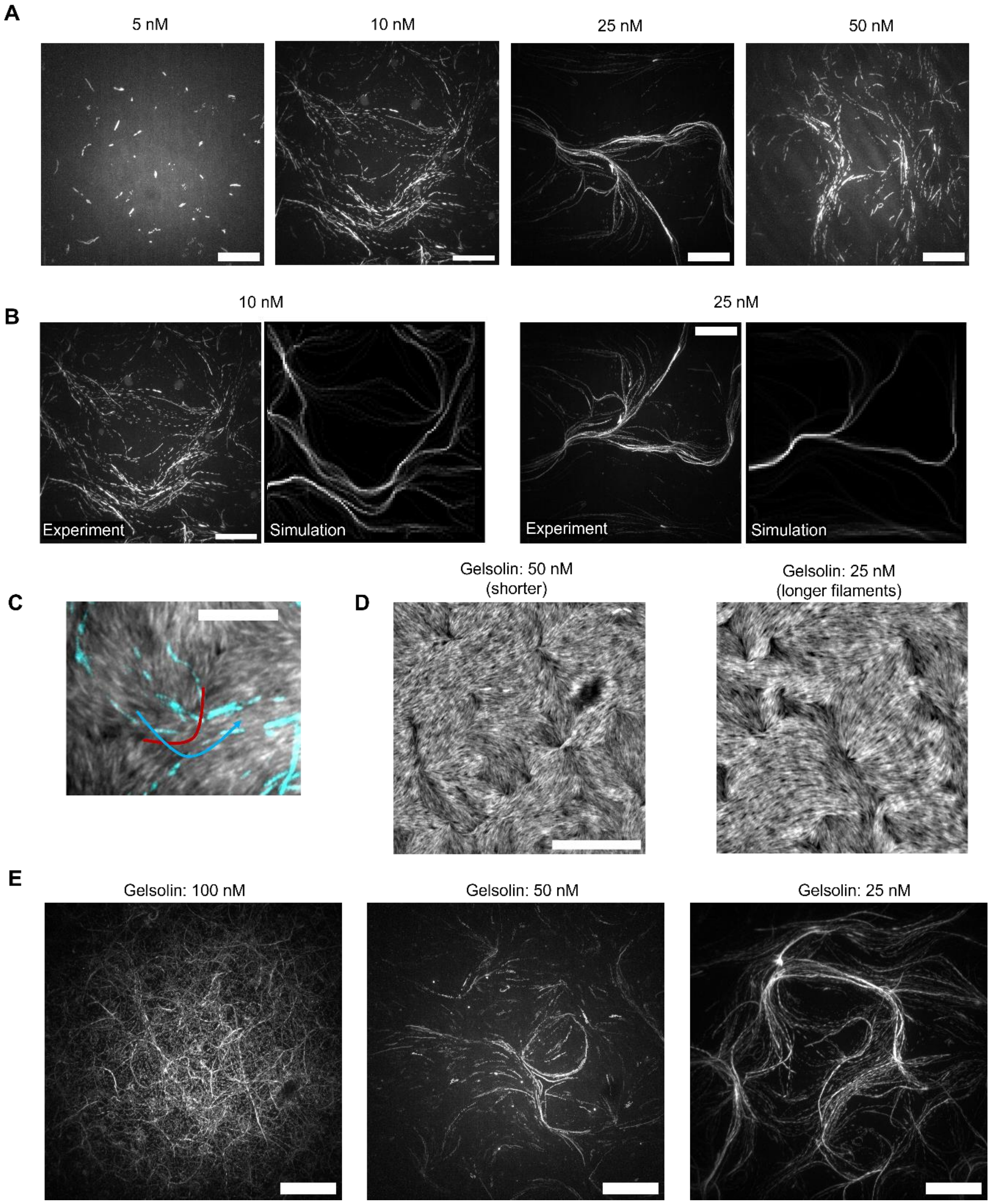

Supplementary* Figure *6. *A) Effect of motors' concentration: Time projection of approximately 20 minutes of recording with different concentration of kinesin motors. In the presence of a nematic, polar patterns are observed at all motors concentrations above 5 nM. Below, while still able to move in the absence of actin, filaments are instead unable to move efficiently inside the nematic. They hence do align with the actin but do not travel much. Scale bars are 25 µm. B) Comparison between simulations (A=0.02 rad/s) and experiments for 10 nM (left) and 25 nM (right) of motors. The model still predicts the formation of polar streams with good accuracy at 25 nM, slightly worse at 10 nM. C) Proof that filaments can escape defects even at 10 nM motors. Time projection of a 4 minutes time series of microtubules in the presence of a +1/2 defect. Microtubules (cyan) are able to escape +1/2 defects in the actin (gray). Scale bar is 5 µm. C) Snapshots of the nematic formed at two different filaments' length, obtained by varying the actin filaments' length using varying concentrations of gelsolin. The higher the gelsolin*

concentration, the shorter the actin filaments. The pictures show slight differences in the number of defects and in their shape. Scale bars are 25 µm. D) Effect of actin filaments length on the formation of patterns. For short filaments (100 nM Gelsolin) no nematic forms in the experimental conditions (1 µM actin, 0.25 % methylcellulose) and no pattern form. For longer filaments when a nematic forms, patterns are always observed if filaments are short enough that microtubules can escape defects (see Fig. S5-7). Scale bars are 25 µm.

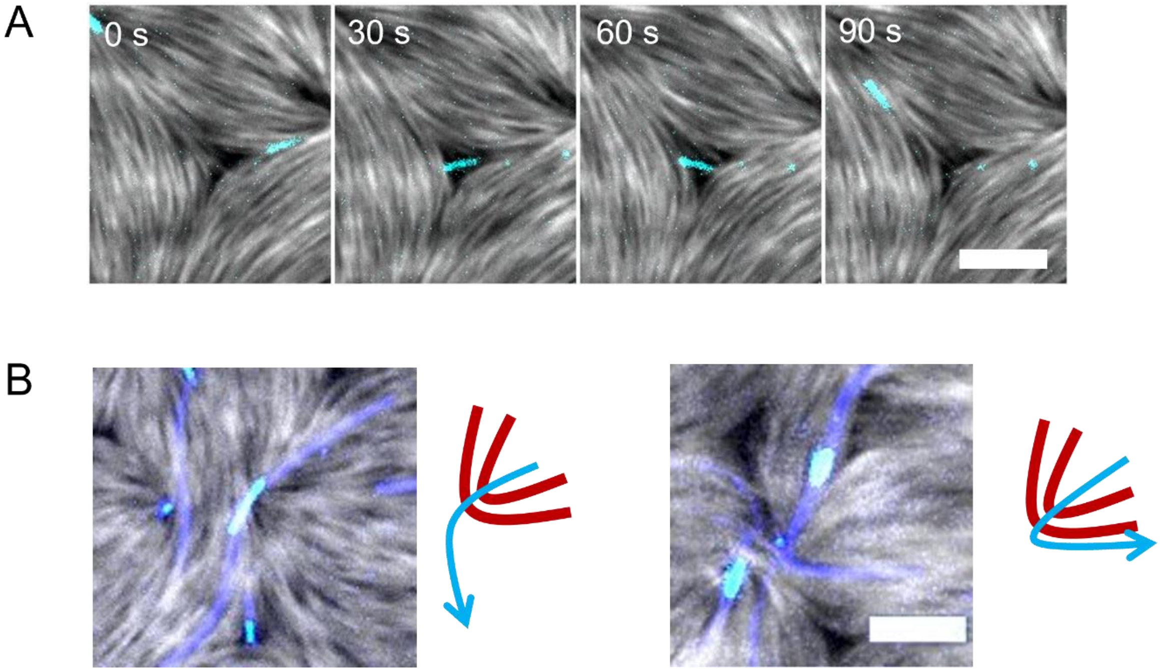

***Supplementary* Figure 7.** *A) MTs occasionally reach the core of a -1/2 defect and only afterwards realign with the nematic. B) Changing the nematic composition (left: Actin length 0.25µm, right 1.5µm) affects the behavior of MTs at defects. Longer actin filaments trap the MTs more efficiently. However, the general behavior is unchanged, with MTs leaving the defect and aligning. Scale bars are 5 µm*

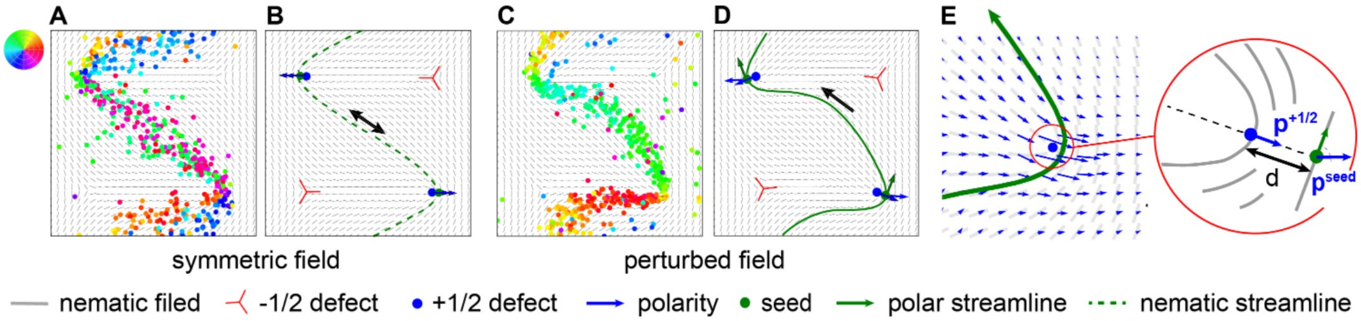

Supplementary Figure 8. Panels (**A**,**C**) show the results of agent-based simulations for symmetric (**A**) and disturbed (**C**) nematic textures. Only in the latter case does polar flow arise, as indicated by the color-coded orientation of particles as shown on the top left. Instead, (**B**) and (**D**) illustrate the heuristic predictions for (**A**) and (**C**), respectively. (**E**) depicts the heuristic rule. Red tripods are for $-1/2$ defects, bold blue dots corresponds to $+1/2$ defects, green lines represents the streams of particles, blue arrows show the divergence of nematic field.

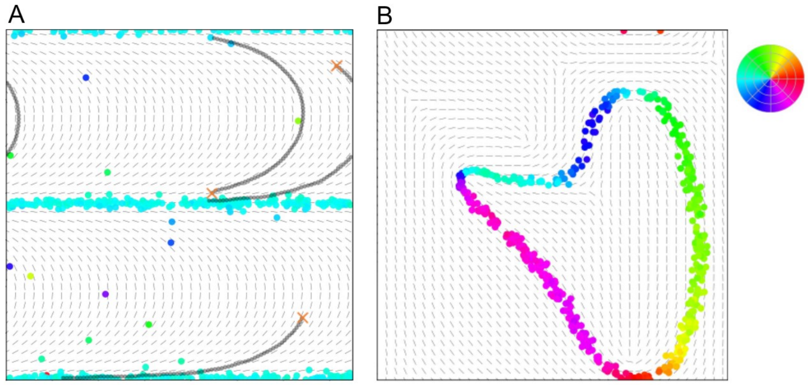

Supplementary Figure 9. Polar lanes in agent-based simulations. **(A)** After agents have been randomly placed in an artificial nematic field with very strong splay; they immediately concentrate into polarly moving lanes. To visualize individual agents, we trace the trajectories of three particles (with their starting position indicated by red crosses). **(B)** Agents form a dense polar ring around three $+1/2$ defects c.f. Fig. 3C of the main text. ($L_1 = 0.1$, $\alpha = 0.1$, $\beta = 0.05$, field resolution of 150×150, $A = 0.8$ and $L_p = 100nm$) (Directional color-code for both panels on the upper right.)

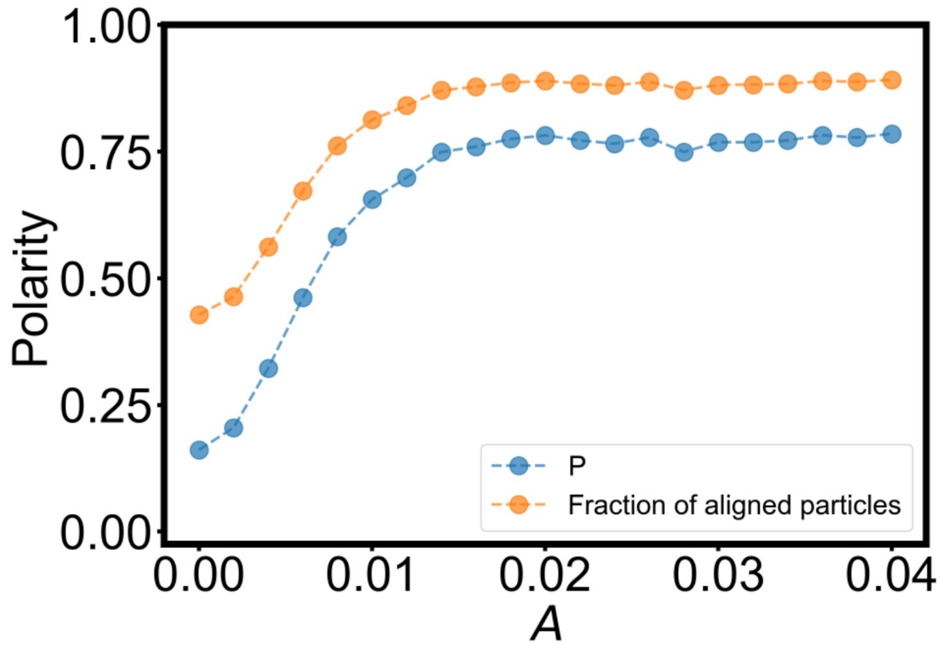

Supplementary Figure 10. Polarity of streams: as A is increased, the total local polar order P strongly increases (blue dots) and more than 90% of the particles eventually move in the polar direction set by the mean flow (orange dots).

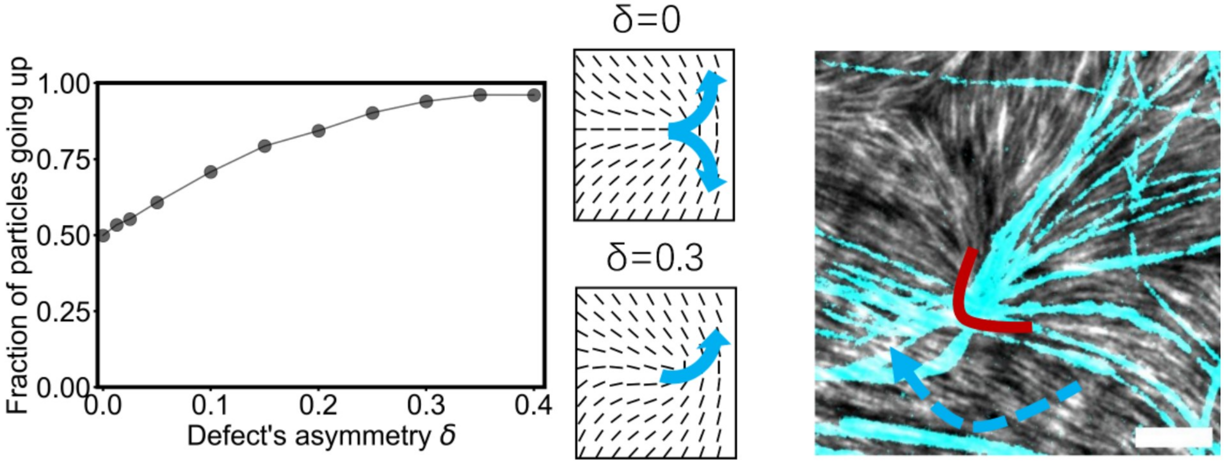

Supplementary Figure 11. Effect of asymmetries in the defect's shape: Left) Polarity of particles, defined as the fractionwe of particles going in one direction (up, in this case) after crossing the defect. As the asymmetry δ is increased, the symmetry breaks and most particles go in one direction. Center) Snapshots of the nematic field at δ=0 and δ=0.3. Blue arrows indicate the mean flow. The formation of polar streams is directly an effect of the asymmetry in +1/2 defects. Right) Time projection of a +1/2 defects at which 93% of the filaments (14 over 15) went in one direction without ever interacting with each other and regardless of the way they entered the +1/2 defect. Dashed blue line indicates mean direction of motion, red line indicates the position of the defect. Scale bar is $5\mu m$.

.

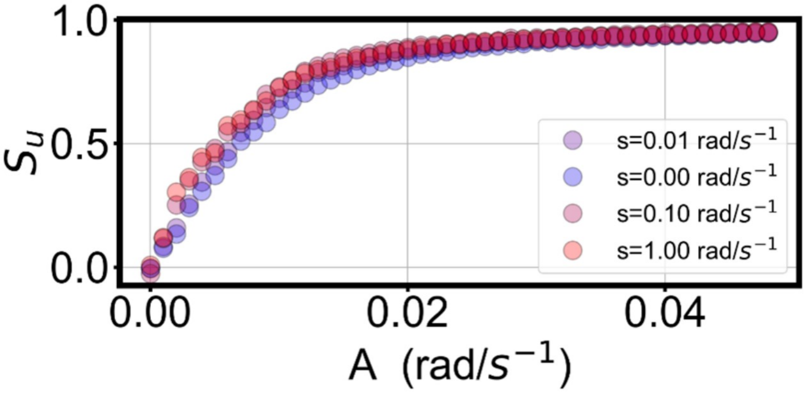

Supplementary Figure 12. Effect of interactions: Changing the parameter s controlling alignment between self-propelled agents (see equation S2) does not affect the results, proving that particle-particle interactions are not responsible for the formation of streams. Independent of the value of s, agents always align with the nematic in the same way as the coupling A is increased, as quantified by the order parameter S_u.

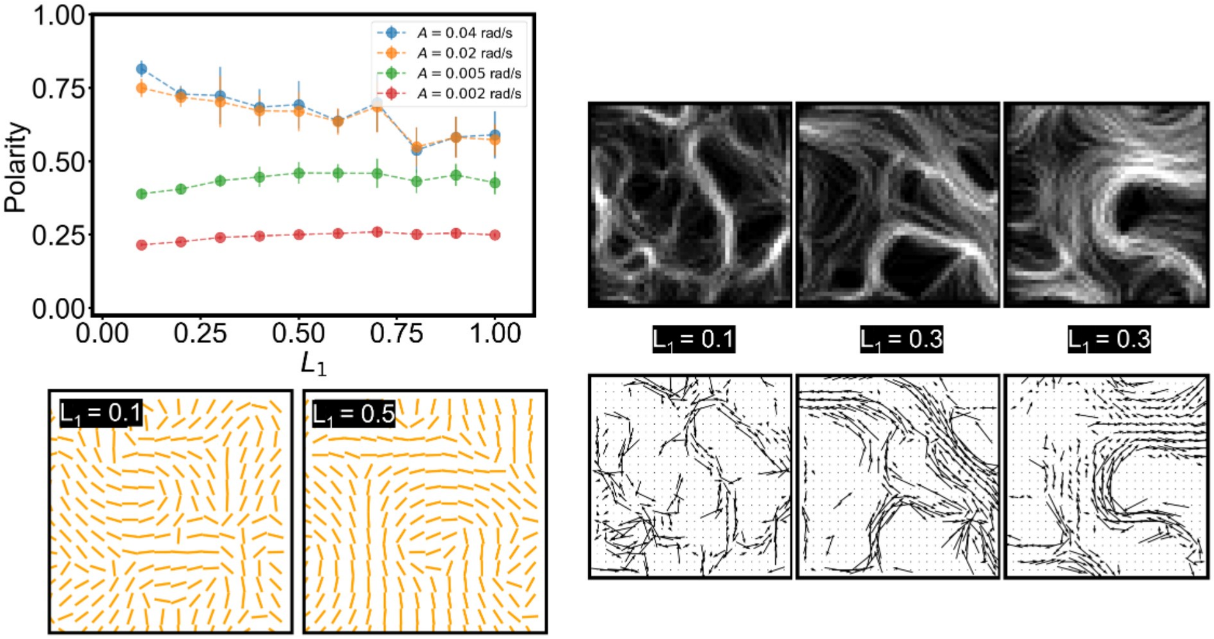

Supplementary Figure 13. Effect of nematic's elasticity: left, top) Polarity (Eq. S9) as a function of the elasticity constant of the nematic field L_1 for different values of the alignment strength A. Data are averaged over 5 independent runs on different nematic fields. As K increases, a small decrease in polarity is observed. Left, bottom) Snapshots of the fields at $L_1 = 0.1$ and $L_1 = 0.5$. Visually, differences are tiny but more rigid fields tend to be less disordered. Right) Exemplary snapshots (top) and flow patterns (bottom) of simulations for $A = 0.02$ and $L_1 = 0.1$, 0.3 and 0.5. Patterns are still locally polar but become wider. The field-of-view size is the equivalent of 50 μm.

.

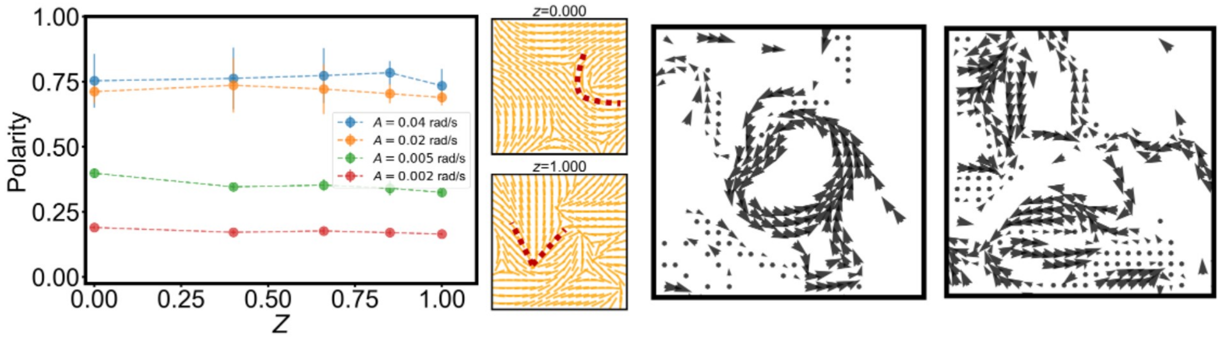

Supplementary Figure 14. Effect of variations in the splay-to-bend ratio z which in turns affect defect's shape. Left) Polarity for different values of A as z is varied. Data are averaged over 5 independent runs on different nematic fields, data is shown as mean +/- standard deviation. No noticeable effect is observed as splay and bend are varied. Center) Snapshots of defects for $z = 0$ and $z = 1$. As z increases, defects become more "pointy" (splay-dominated) but no effect on the formation of polar lanes is observed. Red lines are a guide to the eye that indicate the different defect shape. Right) Mean flow of simulated particles for $z = 0$ (left) and $z = 1$ (right). Polar streams are always observed.

.

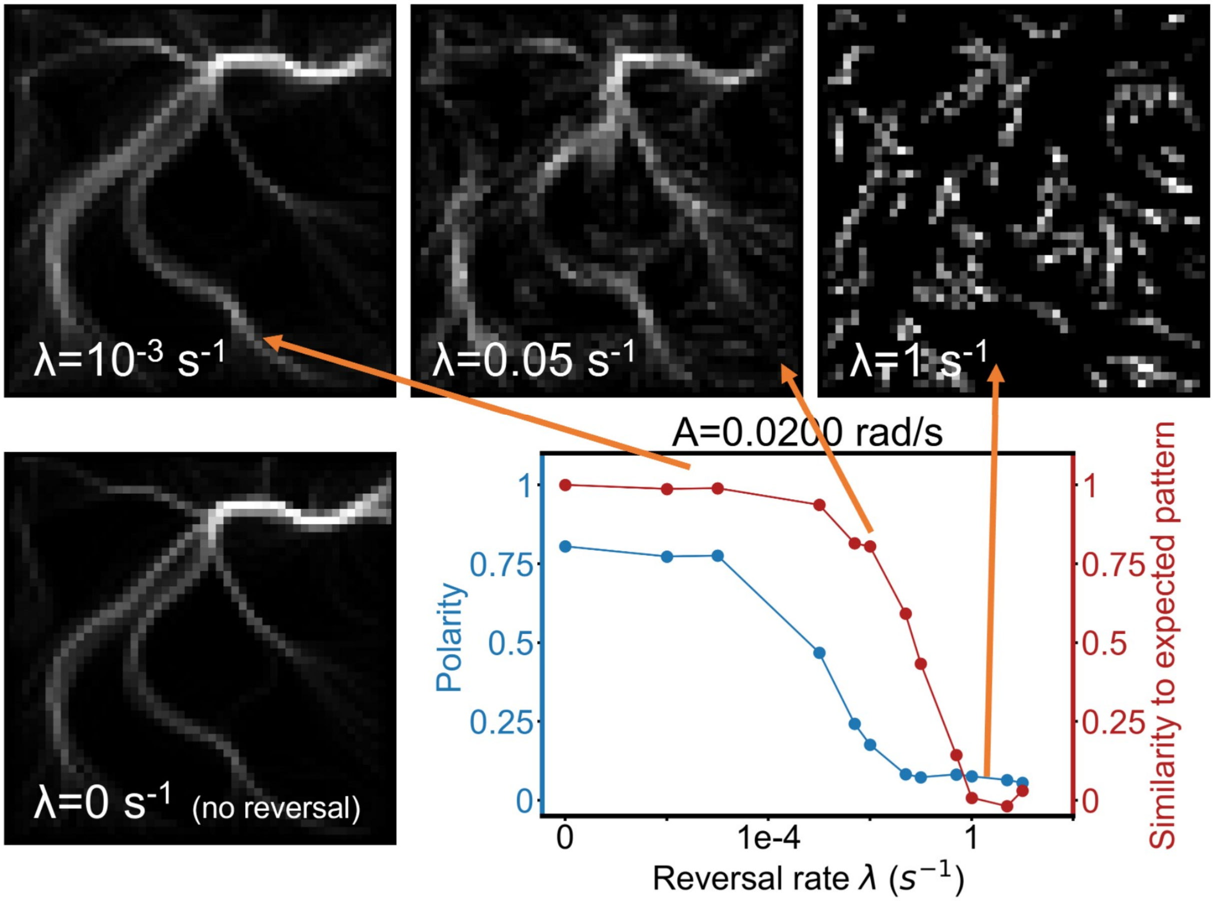

Supplementary Figure 15. Effect of the reversal rate λ: Effect of the reversal rate on the formation of patterns. Snapshots show the mean density of microtubules for different values of the reversal rate λ. As λ is increased, patterns disappear while the density still is inhomogeneous due to the rectification effect due to defects. The plot measures the polarity (measured as the magnitude of the average velocity of particles crossing a given position) and the similarity (measured by the Pearson correlation coefficients with respect to λ=0) as λ is varied. Strikingly, patterns form while $\lambda \gg 0$, but they do not become strongly polar until λ is low enough that particles explore the nematic persistently.

[1] R. Zhang, N. Kumar, J. L. Ross, M. L. Gardel, and J. J. de Pablo, Interplay of structure, elasticity, and dynamics in actin-based nematic materials, Proceedings of the National Academy of Sciences **115**, E124 (2018), arXiv:1709.04961.
[2] M. M. Genkin, A. Sokolov, O. D. Lavrentovich, and I. S. Aranson, Topological defects in a living nematic ensnare swimming bacteria, Physical Review X **7**, 011029 (2017).
[3] I. Maryshev, A. Morozov, A. B. Goryachev, and D. Marenduzzo, Pattern formation in active model c with anchoring: bands, aster networks, and foams, Soft Matter **16**, 8775 (2020).
[4] M. L. Blow, S. P. Thampi, and J. M. Yeomans, Biphasic, lyotropic, active nematics, Phys. Rev. Lett. **113**, 248303 (2014).
[5] A. J. Vromans and L. Giomi, Orientational properties of nematic disclinations, Soft matter **12**, 6490 (2016).
[6] S. Ramaswamy, R. A. Simha, and J. Toner, Active nematics on a substrate: Giant number fluctuations and long-time tails, EPL (Europhysics Letters) **62**, 196 (2003).
[7] R. A. Simha and S. Ramaswamy, Hydrodynamic fluctuations and instabilities in ordered suspensions of self-propelled particles, Physical review letters **89**, 058101 (2002).
[8] D. J. Pearce and K. Kruse, Properties of twisted topological defects in 2d nematic liquid crystals, arXiv preprint arXiv:2104.11293 (2021).
[9] X. Tang and J. V. Selinger, Theory of defect motion in 2d passive and active nematic liquid crystals, Soft Matter **15**, 587 (2019).
[10] T. Turiv, R. Koizumi, K. Thijssen, M. M. Genkin, H. Yu, C. Peng, Q.-H. Wei, J. M. Yeomans, I. S. Aranson, A. Doostmohammadi, et al., Polar jets of swimming bacteria condensed by a patterned liquid crystal, Nature Physics **16**, 481 (2020).

[11] K. Schiele and S. Trimper, On the elastic constants of a nematic liquid crystal, physica status solidi (b) **118**, 267 (1983).
