# Supplementary material for: Polarity and chirality control of an active fluid by passive nematic defects
Source: Nat Mater. 2022 Dec 30;22(2):260–8. doi: 10.1038/s41563-022-01432-w (PMC9894751; doi:10.1038/s41563-022-01432-w)
Supplement: Supplementary file 2 — Reporting Summary [file 41563_2022_1432_MOESM2_ESM.pdf]

nature portfolio

Corresponding author(s): Andreas R. Bausch

Last updated by author(s): Oct 21, 2022

Reporting Summary

Nature Portfolio wishes to improve the reproducibility of the work that we publish. This form provides structure for consistency and transparency in reporting. For further information on Nature Portfolio policies, see our Editorial Policies and the Editorial Policy Checklist.

Statistics

For all statistical analyses, confirm that the following items are present in the figure legend, table legend, main text, or Methods section.

n/a	Confirmed	
☐	☒	The exact sample size (n) for each experimental group/condition, given as a discrete number and unit of measurement
☐	☒	A statement on whether measurements were taken from distinct samples or whether the same sample was measured repeatedly
☒	☐	The statistical test(s) used AND whether they are one- or two-sided<br>*Only common tests should be described solely by name; describe more complex techniques in the Methods section.*
☒	☐	A description of all covariates tested
☒	☐	A description of any assumptions or corrections, such as tests of normality and adjustment for multiple comparisons
☐	☒	A full description of the statistical parameters including central tendency (e.g. means) or other basic estimates (e.g. regression coefficient) AND variation (e.g. standard deviation) or associated estimates of uncertainty (e.g. confidence intervals)
☒	☐	For null hypothesis testing, the test statistic (e.g. F, t, r) with confidence intervals, effect sizes, degrees of freedom and P value noted<br>*Give P values as exact values whenever suitable.*
☒	☐	For Bayesian analysis, information on the choice of priors and Markov chain Monte Carlo settings
☒	☐	For hierarchical and complex designs, identification of the appropriate level for tests and full reporting of outcomes
☐	☒	Estimates of effect sizes (e.g. Cohen's d, Pearson's r), indicating how they were calculated

Our web collection on statistics for biologists contains articles on many of the points above.

Software and code

Policy information about availability of computer code

Data collection	All data as been collected using Leica LAS-X (version 3.7.4.23463) software and a TIRF microscope
Data analysis	Data analysis, processing and simulations have been performed with custom made Python3 scripts and Fiji.

For manuscripts utilizing custom algorithms or software that are central to the research but not yet described in published literature, software must be made available to editors and reviewers. We strongly encourage code deposition in a community repository (e.g. GitHub). See the Nature Portfolio guidelines for submitting code & software for further information.

Data

Policy information about availability of data

All manuscripts must include a data availability statement. This statement should provide the following information, where applicable:

- Accession codes, unique identifiers, or web links for publicly available datasets
- A description of any restrictions on data availability
- For clinical datasets or third party data, please ensure that the statement adheres to our policy

Custom scripts and raw data necessary to reproduce the results, including the source files of movies and images, are available on a Zenodo repository (DOI: 10.5281/zenodo.7071792) and in the Supporting Information. Additional data and code supporting the findings are available from the Corresponding Author.

Human research participants

Policy information about studies involving human research participants and Sex and Gender in Research.

Reporting on sex and gender	n/a
Population characteristics	n/a
Recruitment	na/a
Ethics oversight	n/a

Note that full information on the approval of the study protocol must also be provided in the manuscript.

Field-specific reporting

Please select the one below that is the best fit for your research. If you are not sure, read the appropriate sections before making your selection.

☒ Life sciences ☐ Behavioural & social sciences ☐ Ecological, evolutionary & environmental sciences

For a reference copy of the document with all sections, see nature.com/documents/nr-reporting-summary-flat.pdf

Life sciences study design

All studies must disclose on these points even when the disclosure is negative.

Sample size	No sample size calculation where performed, the samples consist of different microscopy movies and/or different microtubule trajectories (such numbers are indicated in the text). When data has been binned (e.g. Fig2C, flow analysis), the sample size was dependent on the number of elements inside each bin and described by its mean +/- standard deviation. Bins where chosen so that enough data was falling in each bin. In most cases, anyways, as the description is qualitative rather than quantitative, sample size is not particularly relevant in this study.
Data exclusions	No data has been excluded.
Replication	All experiments are performed three times per condition (low and high surface density). From each experiment, 4 different positions are recorded. At each position and for each experiment the nematic field is different, but polar streams are observed in all cases.
Randomization	No randomization was performed as not relevant. Acquired data was divided according to a single experimental parameter (microtubule density) for clarity but the measured effect is not statistical in nature.
Blinding	Blinding is not relevant to this study as no control group is present in the study. No observer bias is relevant to this study.

Reporting for specific materials, systems and methods

We require information from authors about some types of materials, experimental systems and methods used in many studies. Here, indicate whether each material, system or method listed is relevant to your study. If you are not sure if a list item applies to your research, read the appropriate section before selecting a response.

Materials & experimental systems

n/a	Involved in the study	
☒	☐	Antibodies
☒	☐	Eukaryotic cell lines
☒	☐	Palaeontology and archaeology
☒	☐	Animals and other organisms
☒	☐	Clinical data
☒	☐	Dual use research of concern

Methods

n/a	Involved in the study	
☒	☐	ChIP-seq
☒	☐	Flow cytometry
☒	☐	MRI-based neuroimaging
